# SOX11 is a novel binding partner and endogenous inhibitor of SAMHD1 ara-CTPase activity in mantle cell lymphoma

**DOI:** 10.1182/blood.2023022241

**Published:** 2024-01-18

**Authors:** Mohammad Hamdy Abdelrazak Morsy, Ingrid Lilienthal, Martin Lord, Magali Merrien, Agata Magdalena Wasik, Marta Sureda-Gómez, Virginia Amador, Henrik J. Johansson, Janne Lehtiö, Beatriz Garcia-Torre, Jose Ignacio Martin-Subero, Nikolaos Tsesmetzis, Sijia Tao, Raymond F Schinazi, Baek Kim, Agnes L Sorteberg, Malin Wickström, Devon Sheppard, Georgios Z Rassidakis, Ian A Taylor, Birger Christensson, Elias Campo, Nikolas Herold, Birgitta Sander

**Affiliations:** 1 Department of Laboratory Medicine, Division of Pathology, Karolinska Institutet and Karolinska University Hospital, SE14186, Stockholm, Sweden; 2 Department of Applied Medical Chemistry, Medical Research Institute, Alexandria University, 21561, Alexandria, Egypt; 3 Childhood Cancer Research Unit, Department of Women’s, and Children’s Health, Karolinska Institutet, Solna, Sweden; 4 Department of Pharmaceutical Biosciences, Immuno-oncology, Uppsala University Biomedical Centre (BMC), SE-751 24, Uppsala, Sweden; 5 Institut d'Investigacions Biomèdiques August Pi Sunyer (IDIBAPS), Barcelona, Spain; 6 Centro de Investigación Biomédica en Red de Cáncer (CIBERONC), Madrid, Spain; 7 Department of Oncology-Pathology, Karolinska Institutet, Stockholm, Sweden; 8 Institució Catalana de Recerca i Estudis Avançats (ICREA), Barcelona, Spain; 9 Center for ViroScience and Cure, Department of Pediatrics, School of Medicine, Emory University, Atlanta, USA; 10 Macromolecular Structure Laboratory, The Francis Crick Institute, London, United Kingdom; 11 Department of Hematopathology, The University of Texas MD Anderson Cancer Center, Houston, TX, USA; 12 Hematopathology Section, Department of Anatomic Pathology, Hospital Clinic Barcelona, University of Barcelona, Barcelona, Spain; 13 Paediatric Oncology, Astrid Lindgren Children’s Hospital, Karolinska University Hospital, Stockholm, Sweden

**Keywords:** SAMHD1, SOX11, cytarabine, hydroxyurea, MCL

## Abstract

The sterile alpha motif and histidine-aspartate (HD) domain containing protein 1 (SAMHD1) is a deoxynucleoside triphosphate triphosphohydrolase with ara-CTPase activity that confers cytarabine (ara-C) resistance in several haematological malignancies. Targeting SAMHD1’s ara-CTPase activity has recently been demonstrated to enhance ara-C efficacy in acute myeloid leukemia. Here, we identify the transcription factor SRY-related HMG-box containing protein 11 (SOX11) as a novel direct binding partner and first known endogenous inhibitor of SAMHD1. SOX11 is aberrantly expressed not only in mantle cell lymphoma (MCL), but also in some Burkitt lymphomas. Co-immunoprecipitation of SOX11 followed by mass spectrometry in MCL cell lines identified SAMHD1 as the top SOX11 interaction partner which was validated by proximity ligation assay. *In vitro*, SAMHD1 bound to the HMG box of SOX11 with low-micromolar affinity. *In situ* crosslinking studies further indicated that SOX11-SAMHD1 binding resulted in a reduced tetramerization of SAMHD1. Functionally, expression of SOX11 inhibited SAMHD1 ara-CTPase activity in a dose-dependent manner resulting in ara-C sensitization in cell lines and in a SOX11-inducible mouse model of MCL. In SOX11-negative MCL, SOX11-mediated ara-CTPase inhibition could be mimicked by adding the recently identified SAMHD1 inhibitor hydroxyurea. Taken together, our results identify SOX11 as a novel SAMHD1 interaction partner and its first known endogenous inhibitor with potentially important implications for clinical therapy stratification.

## Introduction

Mantle cell lymphoma (MCL) is a rare and aggressive form of non-Hodgkin’s lymphomas with median overall survival of 5 years.^1, 2^ Recently, several new therapeutic strategies including non-covalent Bruton’s Tyrosine Kinase inhibitors (BTKis), Bispecific antibodies and next generation chimeric antigen receptor-T cell therapy (CAR-T) have been developed and shown promising results in the relapsed/refractory settings.^3, 4^

Intensified first-line regimens containing cytarabine (ara-C), followed by consolidating high-dose therapy and autologous stem cell transplantation (ASCT) have significantly improved treatment outcome of MCL.^5, 6^ However, relapses occur after ASCT and MCL remains incurable in most cases.^7, 8^ High-dose ara-C confers durable response to rituximab-based immunochemotherapies and overcome resistance in younger and elderly MCL patients.^5, 9, 10^

Response to ara-C is regulated by the Sterile alpha motif and HD domain containing protein 1 (SAMHD1).^11^ SAMHD1 harbors a dNTP triphosphohydrolase (dNTPase) activity that limits the availability of endogenous dNTPs during G1 phase of the cell cycle and in terminally differentiated cells.^12, 13^ SAMHD1 also hydrolyzes the active triphosphate metabolite of cytarabine (ara-C) known as ara-CTP, limiting its intracellular concentration.^11^ SAMHD1 has been shown to be responsible for ara-C resistance in several haematological malignancies, including AML.^14, 15^ Accordingly, clinical outcome in ara-C treated AML patients is negatively correlated with SAMHD1 expression levels.^15–17^ Contrary to AML, no clear correlation of SAMHD1 expression and ara-C responses could be identified in MCL^18, 19^, suggesting the existence of SAMHD1-modulating factors in MCL.

The transcription factor SRY-related HMG-box containing protein 11 (SOX11), a member of the C family of SOX proteins^20^, is expressed in the majority of conventional MCL cases^21–23^ and a subset of Burkitt lymphomas.^24^ SOX11 is not expressed in normal B cells and does not have a known function dedicated to B-lymphopoiesis.^25–27^ Several lines of evidence have reported an oncogenic role for SOX11 in MCL pathogenesis through regulation of gene expression^2, 28–31^ and augmentation of aberrant B-cell receptor (BCR) signaling.^32^ Aberrant expression and context-specific oncogenic functions of SOX11 have also been described in carcinomas including breast and lung cancers^20, 33–35^. However, the interactome of the SOX11 protein is largely unknown.

Here, we demonstrate that SOX11 directly binds to SAMHD1 via its HMG domain, reduces SAMHD1 tetramerization, impairs the ara-CTPase activity of SAMHD1 and confers enhanced sensitivity to ara-C in MCL, both *in vitro* and *in vivo* models.

## Methods

### Cell lines and culture

The MCL cell lines Granta-519, JeKo-1 and JVM-2 were purchased form the German collection of microorganisms and cell culture (DSMZ), Germany. Rec1 was a kind gift from Dr. Christian Bastard. Cells were cultured in RPMI 1640-Glutamax (Gibco, life technologies, UK) supplemented with 10% heat-inactivated fetal bovine serum (FBS) (Gibco, life technologies, UK) and 50 μg/mL Gentamicin (Gibco, life technologies, UK) and maintained at 37°C and 5% CO_2_ and split every three days to a density of 0.5x10^6^ cells/mL. Doxycycline-inducible JVM-2 cells ectopically overexpressing SOX11 (JVM-2^iSOX11^) and its control (JVM-2^vector^) were also included in this study. (See also Supplemental information)

## Ethical Approval

The study has been performed in accordance with the Declaration of Helsinki, including informed patient consent, and has been approved by the Ethical Committee in Stockholm 2018/2182–32. Animal experiments were approved by the regional animal ethics committee of Stockholm County (approval 13820-2019) in accordance with the Animal Protection Law (SFS1988:534), the Animal Protection Regulation (SFS 1988:539) and the Regulation for the Swedish National Board for Laboratory Animals (SFS1988:541).

### Primary MCL cells

Cryo-preserved cells taken from diagnostic samples of patients with MCL from our recent study^19^ were used for SOX11-SAMHD1 colocalization by proximity ligation assay. Three samples with high lymphoma cell purity (>90% of MCL cells) were selected for ara-C treatment and labelled as PS1-3. For details, see Supplemental Table 1.

### Genetic silencing of SOX11 by siRNA

Small interfering RNA (siRNA) experiments were performed using predesigned siRNA against *SOX11* (4392420, Ambion), and scramble non-targeting siRNA (4390844, Ambion) was used as negative control. Granta519 and JeKo-1 cells were maintained at a density of 0.5x10^6^ cells/mL 24 hours prior to transfection with siRNAs. The desired number of cells to be transfected was resuspended in 100 μL of nucleofection reagents supplied by Amaxa Cell line Nucleofector kit C (VCA-1004, LONZA), containing 1 μM of the respective siRNA, electroporated using AMAXA machine, program X-01 and immediately fed by warm RPMI 1640 containing 20% FBS, and maintained at 37°C and 5% CO_2_ for 48 h.

### Viral-like particle-mediated depletion of SAMHD1

The ablation of SAMHD1 at the protein level was carried out using inactivated viral-like particles (VLPs) including Vpx which targets SAMHD1 for ubiquitin-mediated proteolysis. The VLPs were provided and prepared as previously described^17^ and the references therein. A non-targeting particle (dX) was used as negative control. The efficiency of SAMHD1 depletion was validated by Western blotting as described below.

### Treatment

Cytosine-b-D-arabinofuranoside (C1768-100MG) was purchased from Sigma and dissolved in RPMI 1640 into final concentration of 10 mg/ml. Hydroxyurea (H8627) purchased from Sigma was also used in combination to cytarabine (See Supplemental information)

### Cell cycle analysis

Analysis of the cell cycle was performed using the propidium iodide (PI) flow cytometry kit (ab139418, Abcam, Netherlands), according to the manufacturer’s protocol. (See Supplemental information)

### Western blotting

Protein expression was measured by performing Western blotting using total cell lysates from siRNA-transfected, Vpx-treated, SOX11-induced or ara-C treated cell lines as previously described.^19^ Blots were developed using Western Lightning Plus-ECL, Enhanced Chemiluminescence Substrate (NEL104001EA) and visualization and semi-quantification were performed using LiCOR machine and Odessey software, respectively. For re-probing purposes, membranes were stripped using Restore Western Blot Stripping Buffer (Thermofisher, cat#21059). Information regarding manufacturer and dilution for all antibodies can be found in Supplemental Table2.

### Crosslinking and native gel electrophoresis

To assess tetramerization of SAMHD1, both JVM-2^vector^ and JVM-2^iSOX11^ cultured in doxycycline-supplemented media were crosslinked with a disuccinimidyl glutarate (DSG) (#20593, Thermofisher) at the concentrations 5, 2.5, 1.25, 0.625 and 0.312 mM for 30 min at room temperature as described in a study by Rudd *et al*.^36^ Crosslinking was quenched by addition of Tris 1M pH 8 for 30 minutes at room temperature. Crosslinked cell pellets were washed twice with PBS (1X) and resuspended in RIPA buffer supplemented with protease- and phosphatase-inhibitor cocktail. Samples were quantified and mixed with Laemmli buffer free of β-mercaptoethanol and boiling was omitted before electrophoresis using NuPAGE 4-12% Bis-Tris gels (NP0321BOX, Invitrogen). The rest of the Western blot procedure was performed as described above.

### Heterotopic JVM-2 animal model

Forty-five-day-old female NMRI nu/nu mice (BomTac: NMRI-Foxn1nu, Taconic) were housed with eight mice per cage and given sterile water and food ad libitum. Sample size was estimated to be six animals per group in a total of eight groups for a power of 0.8 and a significance level of 0.05, estimating a hypothetical mean difference in survival of 50% and a standard deviation of 30%. A surplus of two mice per group was used to account for possible xenotransplant failures or other unexpected occurrences. Conditions were first tested in a pilot experiment, with the timing of drug injection determined to be day 5 after cell injection, corresponding to the time when the animals reached a tumor volume of 150 mm^3^.

### Co-IP and mass spectrometry

Co-immunoprecipitation was performed using Granta519 and JeKo1 cells cross-linked (2 x 10^7^ cells per sample) in 11 % formaldehyde solution (11 % formaldehyde, 0.1 M NaCl, 1 mM EDTA, 0.5 mM EGTA, 50 mM Hepes, pH 8). Detailed protocol for Co-IP, sample preparation for mass spectrometry, LC-ESI-LTQ-Orbitrap analysis, peptide and protein identification is explained in Supplemental information. The following steps were used to define SOX11 interacting proteins: 1) filter out protein identifications in any of the 6 IgG control pull downs (3 replicates in each cell line), 2) keep potential SOX11 interacting proteins with identification in all 3 replicates in either or JeKo-1 or Granta, 3) require the mean number of peptide spectrum matches (PSMs) in the three replicates of JeKo-1 or Granta to be >2 PSMs.

### Cell viability assay

Cell viability was assessed using CellTiter 96 AQueous One Solution Cell Proliferation Assay (MTS)-Promega. For one test 20 μL of MTS solution was added to 100 μL of the cultured cells and kept at conditions of 37°C and 5% CO_2_ for 3 h and absorbance at 490 nm was measured using ClarioStar reader (BMGlabtech). Viability values were calculated by normalizing absorbance of treated cell to the absorbance of their respective untreated controls.

For assessing the response of induced JVM-2 cells with different concentrations of doxycycline to cytarabine, we used CellTiter-Glo® Luminescent Cell Viability Assay (Promega). For each test, 50 μL of CellTiter-Glo® reagent were added to equal volume of cell suspension in 96-well plate and kept on a plate shaker for 20 min at room temperature to allow cell lysis. Luminescence was measured using a ClarioStar reader.

### HPLC-MS/MS assay for measurement of intracellular dNTPs and ara-CTP

Both JVM-2^vector^ and JVM-2^iSOX11^ treated with 10 μM ara-C for 24 h were collected, washed with PBS and lysed in 65% methanol at 95°C for 3 minutes. Lysed samples were centrifuged at 13,000 RPM; and supernatants underwent speed vacuum dry for subsequent chromatography–tandem mass spectrometry method.^37^ HPLC-MS/MS is described in ^36^.

### Immunocytochemistry and proximity ligation assay

Immuno-fluorescence staining was performed on 4% paraformaldehyde-fixed and 0.1% Triton-X100-permeabilized cells on Superfrost PlusTM Adhesion slides (ThermoFisher), followed by confocal microscopy imaging. Duolink in situ proximity ligation assay (PLA) was performed using the kit DUO92008 (Merck). See the methods section of the Supplemental information for further details.

### Cellular Thermal Shift Assay (CETSA)

To assess the shift of thermal aggregation temperature of SAMHD1 upon SOX11 overexpression, 1x10^6^ JVM-2^vector^ and JVM-2^iSOX11^ cells were collected and resuspended in 60 μL of TBS buffer (pH 7.5). Cell suspensions were heated at a range of temperature 38, 42, 48, 52, 55 and 60°C for 3 min, followed by 3 min incubation at room temperature. Cells were then lysed by 3 times of freezing/thawing cycles. Each cycle was comprised of 3 min on dry ice followed by 3 min incubation in water bath at 37°C. Total protein was quantified by Bradford assay and the procedure of Western blot was performed as described above. Band intensities of SAMHD1 at the different conditions were normalized to the respective band intensities of the thermostable Superoxide dismutase-1 (SOD-1) and the percentage of remaining proteins were calculated and plotted to sigmoidal Boltzmann curve using GraphPad software (La Jolla California, USA).

### Microscale Thermophoresis

Binding of SOX11 HMG to SAMHD1 was measured using Microscale Thermophoresis (MST). Experiments were performed in 25mM Hepes (pH8.0), 150mM NaCl, 5mM MgCl_2_, 5mM DTT, 0.02% tween-20, and 0.1mg/mL BSA in standard capillaries on a Monolith NT.115. Fluorescence was observed using the Nanotemper His-Tag Red-tris-NTA at 25 nM bound to SAMHD1H215A(1-626) at 100 nM for 10 seconds at 80% MST power and 80% LED power. See the methods section of the Supplemental information for further details.

### Confocal imaging

Imaging was performed using a Nikon A1R confocal laser scanning microscope equipped with an inverted microscope Nikon Eclipse Ti-E. Laser lines used were 405 nm (DAPI), 488 nm (FITC) and 561 nm (TRITC). Images were captured with the imaging software NIS-Elements version 5.30.02. Fluorophores used in this study were FITC and TRITC and DAPI and TEXAS RED. The aperture size (pinhole) of objective lenses was set at 1.2. Images were captured at a magnification of 100X for PLA. For details about image analysis, see the Supplemental information.

### Statistical analysis

Statistical analysis was performed using Graph Prism version 6 (GraphPad software, La Jolla California, USA). The experiments were conducted in at least two independent biological replicates and the data were represented as mean ± SEM with 95% confidence intervals.

### ZIP synergy analysis

After determining full dose-response curves for each drug, drug-drug interaction of drug combinations was determined using a Zero Interaction Potential (ZIP) algorithm, which determines the degree of combination synergy, or antagonism, between drug combinations by comparing observed response against the expected response which assumes no interaction between drugs.^38^

## Results

### SOX11 directly binds to SAMHD1 in MCL

**Figure 1:**
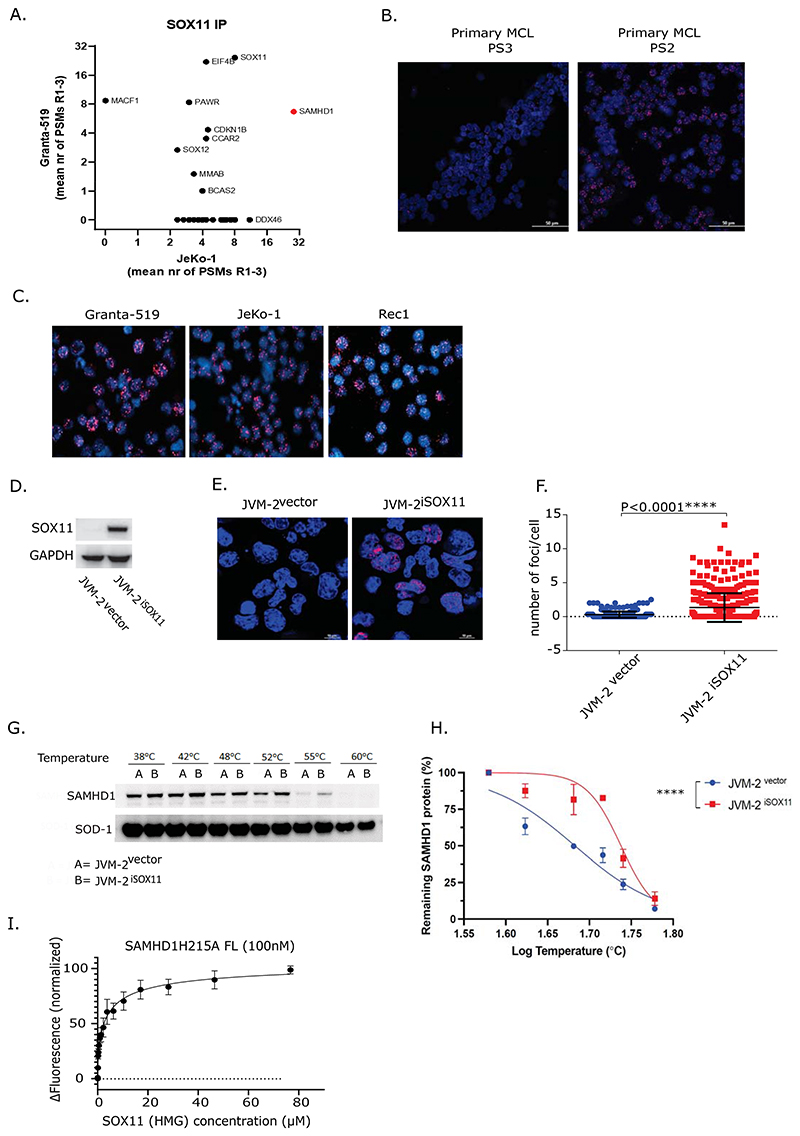
SOX11 binds to SAMHD1 in MCL. A. Identification of SOX11 protein interactors by mass spectrometry-based proteomics analysis of co-immunoprecipitation of SOX1 in Granta-519 and JeKo-1 cell lines. Three biological replicates were analyzed per cell line, and the mean number of peptide spectrum matches (PSMs) across the 3 replicates per protein are displayed on the axes. Proteins with zero PSMs indicate absence of interaction in that cell line. See methods for defining SOX11 interacting proteins. B. Proximity ligation assay performed on two primary MCL cells with different SOX11 expression levels (See Supplemental Table1) using rabbit polyclonal anti-SOX11 and mouse monoclonal anti-SAMHD1. DAPI channel represents stained nuclei, whereas red channel (TRITC) represents SOX11-SAMHD1 colocalization. Magnification 60x, scale bar 50 μm and pinhole is set at 1.2. The red fluorescent foci represent the colocalization between SOX11 and SAMHD1. C. Proximity ligation assay performed on Granta-519, JeKo-1 and Rec1 cells using rabbit polyclonal anti-SOX11 and mouse monoclonal anti-SAMHD1. Magnification 60x and pinhole set at 1.2. D. Representative western blot showing the efficiency of doxycycline-induced expression SOX11 in JVM-2^vector^ and JVM-2^iSOX11^ at 72 h after doxycycline treatment. SOX11 band is detected at 74 KDa and GAPDH at 37 KDa. E. Representative images of proximity ligation assay performed on JVM-2^vector^ and JVM-2^iSOX11^ using rabbit polyclonal anti-SOX11 and mouse monoclonal anti-SAMHD1, magnification 60x, Scale bar 10μm and pinhole set at 1.2. Cells were treated with doxycycline 0.1 μM for 72 h. F. Scatter plot shows number of fluorescent foci per cell. The number of foci per cell were analyzed in total 350 cells of JVM-2^vector^ or JVM-2^iSOX11^ using CellProfiler software. The data are represented as mean ± SEM of three independent biological replicates. *P* <0.0001 was calculated by unpaired, two-tailed t-test with Welch correction. G. CETSA performed using JVM-2^vector^ and JVM-2^iSOX11^ 72 h after treatment with 0.1 μM doxycycline. A representative western blot showing band intensities of SAMHD1 (71 KDa) and thermostable SOD-1 (20 KDa). H. Sigmoidal Boltzmann curve of percentage of remaining SAMHD1 protein on y-axis and log_10_ temperature on x-axis. Data are represented as mean ± SEM of three independent biological replicates. ****, *P* (two-way ANOVA) < 0.0001. I. Binding of SOX11 HMG to SAMHD1 measured by MST. The fluorescence change in labelled SAMHD1 upon titration with SOX11 HMG is plotted, error bars are standard deviation of the mean values from three independent experiments. Fitting of the data to a hyperbolic binding isotherm gives *K*_*D*_ = 3.2 ± 0.6 μM.

To identify potential interaction partners of SOX11, we performed co-immunoprecipitation of nuclear SOX11 in two well-characterized SOX11-positive MCL cells lines, Granta-519 and JeKo-1, and analyzed the recovered proteins by high-performance liquid chromatography/mass spectrometry (HPLC/MS) (Supplemental Figure 1A). SAMHD1 was the top significant partner protein of SOX11 in both cell lines (Figure 1A & Supplemental Figure 1B, see also Supplemental Dataset 1). To validate a possible SOX11-SAMHD1 colocalization, we carried out an *in situ* proximity ligation assay (PLA) in cells of three primary MCL (PS1, PS2 and PS3) with different level of SOX11 expression (Supplemental Table 1) as well as three SOX11-positive MCL cell lines (Granta-519, JeKo-1 and Rec1) using SAMHD1 and SOX11 antibodies (Figure 1B, C & Supplemental Figure 2A-D). To further validate specificity for SOX11, we ectopically expressed SOX11 in SOX11-inducible JVM-2^iSOX11^ cells (Figure 1D), derived from the SOX11-negative MCL cell line JVM-2. As expected, a positive PLA signal was seen only upon induction of SOX11 (Figure 1E-F & Supplemental Figure 3A). Colocalization as assessed by confocal immunofluorescence microscopy was consistent with SOX11-SAMHD1 interaction (Supplemental Figure 3 B, C). Moreover, results of cellular thermal shift assays (CETSA) suggested physical interaction of SAMHD1 and SOX11 as evidenced by the shift of the thermal aggregation temperature of SAMHD1 from 41°C to 54°C (Figure 1G-H) in the absence (JVM-2^vector^) or presence (JVM-2^iSOX11^) of SOX11, respectively. To address whether this interaction was direct, we performed Microscale Thermophoresis (MST) with recombinant SAMHD1 and the high-mobility group (HMG) domain of SOX11. These experiments revealed direct binding of the SOX11-HMG domain to SAMHD1 with low micromolar affinity (*K*_*D*_=3.2±0.6 μM) (Figure 1I). Collectively, these results suggest a direct interaction of SOX11 with SAMHD1.

### SOX11 negatively regulates ara-CTPase activity of SAMHD1

**Figure 2:**
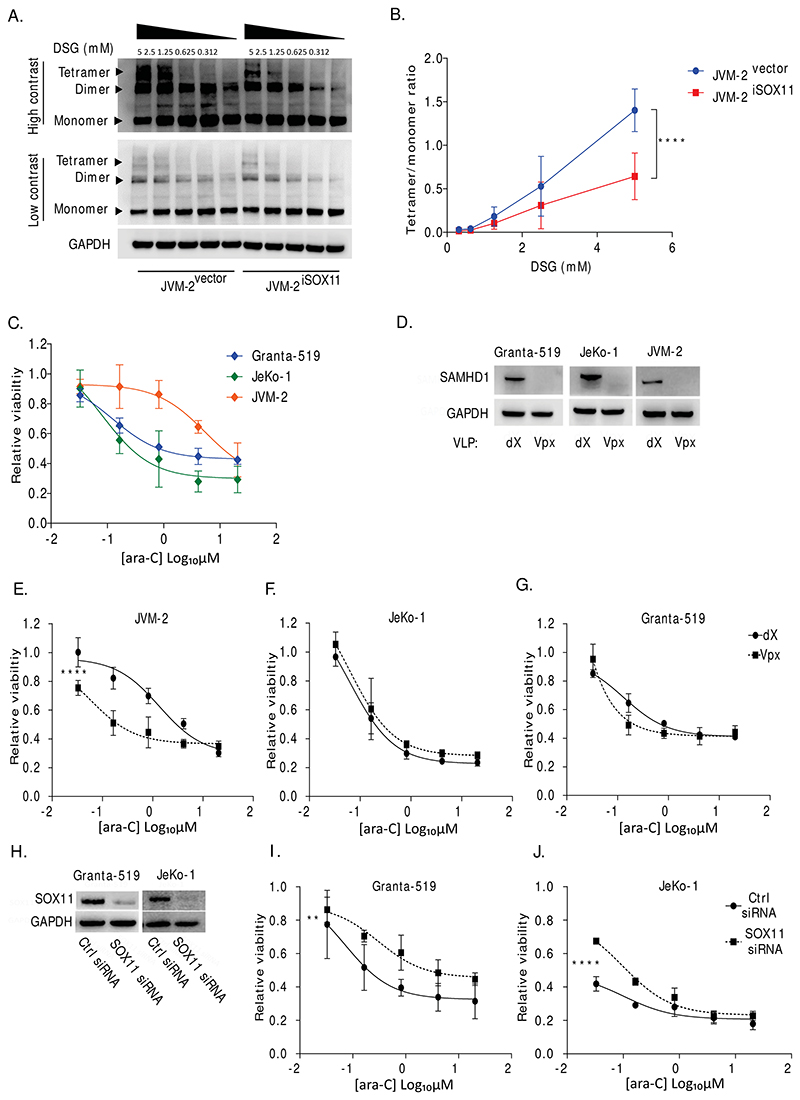
SOX11 impairs tetrameric configuration of SAMHD1 and confers ara-C sensitivity. A. Representative western blot of native gel electrophoresis performed using disuccinimidyl glutarate (DSG)-crosslinked in JVM-2^vector^ and JVM-2^iSOX11^ 96 h after treatment with 0.1 μM doxycycline. The tetrameric form of SAMHD1 is detected at a size of ~250 KDa, Dimer at ~150 KDa, monomer at 71 KDa, and GAPDH at 37 KDa. B. Curve shows tetramer/monomer ratio in crosslinked in JVM-2^vector^ and JVM-2^iSOX11^ with different concentrations of DSG (related to A). Data are represented as mean ± SEM of three independent biological repeats. *P* (two-tailed, two-way ANOVA) is indicated on the curve. C. Dose response curve for ara-C in Granta-519, JeKo-1 and JVM-2 treated for 72 h by CellTiter MTS assay. The values on the y-axis represent the relative viability values which were calculated by normalizing 100% values to respective untreated controls. Data are represented as mean ± SEM of three independent experiments. D. Western blot showing the depleting efficiency of SAMHD1 by Vpx in the three cell lines compared to non-targeting dX. One representative western blot out of three is shown. Cells were treated with dX or Vpx for 3 h before ara-C treatment and cultured thereafter for 72 h before harvesting. SAMHD1 was detected at 71 KDa and GAPDH at 37 KDa. E-G. Dose response curves for ara-C determined in JVM-2 (E), JeKo-1 (F) and Granta-519 (G) with Vpx or dX for 3 h before ara-C treatment. Viability was measured by CellTiter MTS assay after 72 h of ara-C treatment. The values on the y-axis represent the relative viability values which were calculated by normalizing absorbance value at each dose of ara-C for each condition to respective untreated controls. Data are represented as mean ± SEM of three independent biological replicates. *P* (two-tailed) was calculated using two-way ANOVA. **** (*P* < 0.0001) H. Western blot showing the efficiency of *SOX11* silencing in Granta-519 and JeKo-1 by siRNA for 48h. One representative western blot out of two is shown. SOX11 was detected at 74 KDa and GAPDH at 37 KDa. I-J. Dose response curve for 72 h ara-C treatment in non-targeting control siRNA- and SOX11 siRNA-transfected Granta-519 (I) and JeKo-1 (J). ara-C treatment was applied 8 h after transfection. Viability was measured using Celltiter MTS assay after 72 h of ara-C treatment. Data are represented as mean ± SEM of two independent biological replicates. *P* (two-tailed) was calculated using two-way ANOVA. ** (*P* < 0.01), **** (*P* < 0.0001)

As enzymatic activity of SAMHD1 requires allosterically regulated homo-tetramerization^39, 40^, we next investigated the effect of SOX11 on steady-state levels of SAMHD1 homo-oligomers. To this end, native gel electrophoresis of SAMHD1 in JVM-2^iSOX11^ following *in situ* crosslinking showed that SOX11 induction significantly reduced the SAMHD1 tetramer-to-monomer ratio (Figure 2A, B) (*P* < 0.0001). Hence, the SOX11-SAMHD1 interaction has direct effects on the SAMHD1 cellular quaternary configuration.

As a reduction of tetrameric SAMHD1 levels suggested a reduction of SAMHD1’s enzymatic activity, we were prompted to investigate the functional consequences of SOX11-SAMHD1 interaction on ara-C efficacy in MCL using ATP-release based cell proliferation inhibition assays. While comparisons across different cell lines are inherently difficult, SOX11-negative JVM-2 cells exhibited an up to approximately 60-fold higher IC_50_ (5 μM) for ara-C as compared to SOX11-positive JeKo-1 (0.08 μM) and Granta-519 (0.12 μM) (Figure 2C). As all three cell lines express SAMHD1 (Figure 2D), these results suggest a SOX11-mediated SAMHD1 inhibition. Next, we examined the effect of SAMHD1 depletion on ara-C sensitivity by delivering Simian Immunodeficiency Virus protein (Vpx) using non-infectious virus-like particles (VLPs) to target SAMHD1 for ubiquitin-mediated proteasomal degradation^17^ (Figure 2D). SAMHD1 ablation significantly sensitized SOX11-negative JVM-2 to ara-C and shifted the IC_50_ for ara-C by a factor of ~20 as compared to control treatment with VLPs lacking Vpx (dX) (Figure 2E, *P* < 0.0001). However, SAMHD1 depletion had no significant impact on the response to ara-C in SOX11-positive JeKo-1 (*P* = 0.15) or Granta-519 (*P* = 0.32) (Figure 2F, G, respectively). These findings support the notion that the catalytic activity of SAMHD1 is impaired by SOX11.

### SOX11 sensitizes MCL to ara-C by inhibiting SAMHD1

**Figure 3:**
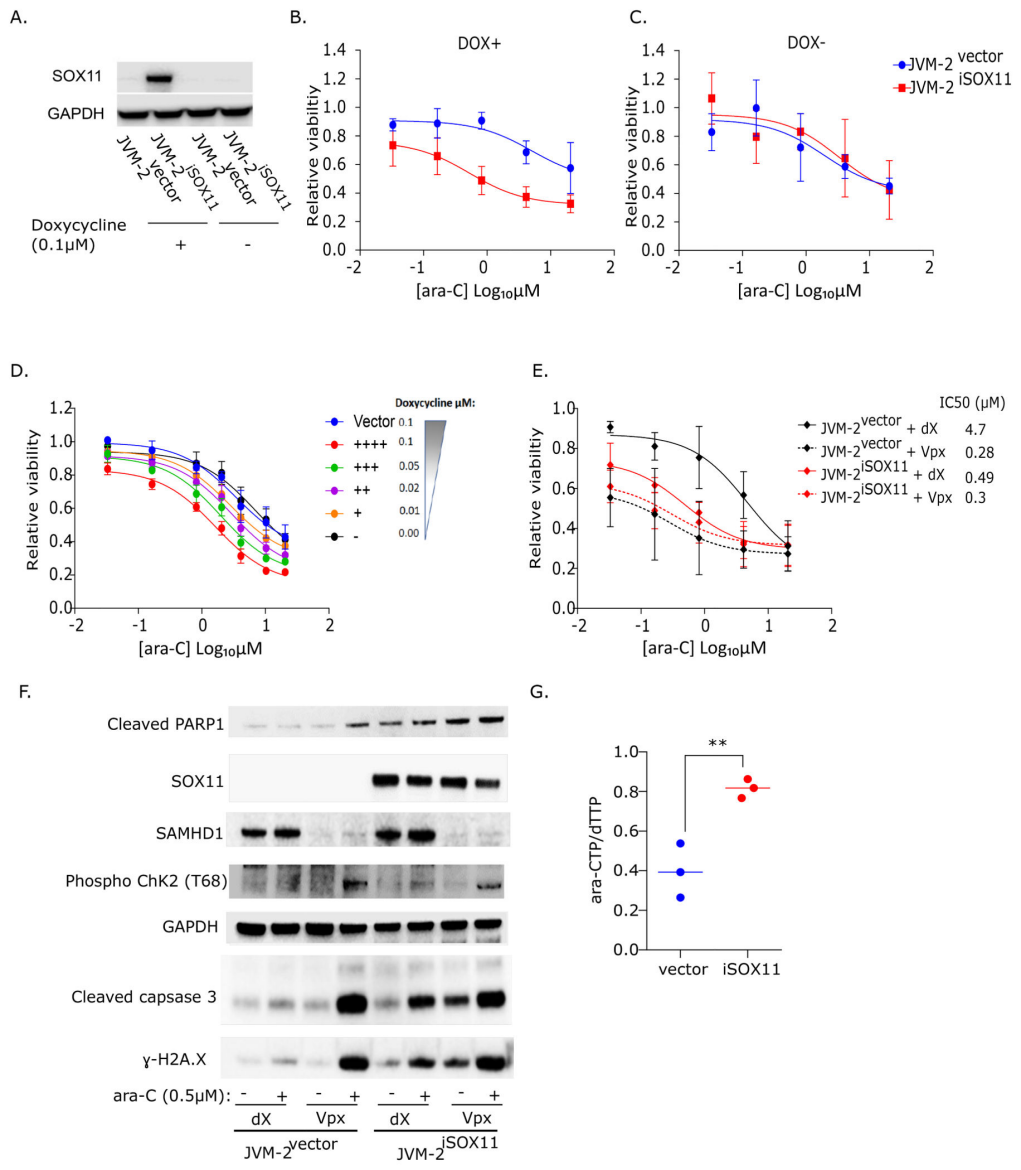
SOX11 expression sensitizes MCL cells to ara-C through impairing ara-CTPase activity of SAMHD1. A. Representative western blot showing SOX11 expression patterns in JVM-2^vector^ and JVM-2^iSOX11^ in presence and absence of 0.1 μM doxycycline for 96 hours. One representative western blot out of six replicates is shown. SOX11 was detected at 74 KDa and GAPDH at 37 KDa. B-C. Dose response curve for ara-C treatment for 72 h in JVM-2^vector^ and JVM-2^iSOX11^ in presence (B, DOX+), and absence (C, DOX-) of doxycycline (DOX). ara-C treatment was applied 24 h after doxycycline (0.1 μM) treatment. Data are represented as mean ± SEM of six independent biological replicates. D. Dose response curve for 72 h treatment with ara-C in JVM-2^vector^ and JVM-2^iSOX11^ cultured in the indicated concentrations of doxycycline. ara-C treatment started 24 h after doxycycline-induced SOX11 expression. Viability was measured using CellTiter-Glo® Luminescent Cell Viability Assay after 72 h of ara-C treatment. The values on the y-axis represent the relative viability values which were calculated by normalizing luminescence value at each dose of ara-C for each condition to respective untreated controls. Data were represented as mean ± SEM of four independent biological replicates. E. Dose response curve for ara-C determined in dX- or Vpx-treated JVM-2^vector^ and JVM-2^iSOX11^ that were induced by 0.1 μM doxycycline. After 24h of culturing in doxycycline-supplemented media, cells were treated with dX or Vpx for 3 h, followed by ara-C treatment for 72 h. Viability was measured using CellTiter MTS assay after 72 h of treatment. The values on the y-axis represent the relative viability values which were calculated by normalizing absorbance values at each dose of ara-C for each condition to respective untreated controls. Data are represented as mean ± SEM for five independent biological replicates. F. Western blot shows the effect of treatment of ara-C at sublethal dose (0.5 μM) on cleaved PARP1 was detected at 89 KDa, phospho-ChK2 (T68) (~62 KDa), cleaved caspase3 (17 KDa) and γ-H2A.X (14 KDa) in dX- or Vpx-treated JVM-2^vector^ and JVM-2^iSOX11^. ara-C treatment was applied after 3 h of treatment with either dX or Vpx and treated cells were harvested after 72 h of ara-C treatment, as explained in A. One representative experiment out of three is shown. G. Intracellular ara-CTP levels normalized to the canonical dTTP, determined using HPLC-MS/MS. Both JVM-2^vector^ and JVM-2^iSOX11^ were treated with 10 μM of ara-C for 24 h. Circles and error bars correspond to individual values, mean ± SEM of at three independent experiments. Analyses were performed using unpaired two-tailed t-tests. **P < 0.01.

Transient downregulation of *SOX11* by RNA interference conferred partial ara-C resistance in Granta-519 and JeKo-1 with an increase of the IC_50_ ~5-fold and ~20-fold, respectively (Figure 2H - J). Conversely, doxycycline-induced SOX11 overexpression in JVM-2^iSOX11^ significantly sensitized cells to ara-C and reduced the IC_50_ ~10-fold from 5 to 0.53 μM (*P* < 0.0001), whereas no effect was seen in JVM-2 vector control cells or non-induced JVM-2^iSOX11^ (Figure 3A-C). Moreover, doxycycline titrations revealed that the extent of ara-C sensitization was dependent on the protein expression level of SOX11 (Figure 3D, Supplemental Figure 4 & Supplemental Figure 5A). Given that ara-C is an S-phase specific drug^41^, we wished to rule out indirect effects of SOX11 on cell cycling and thus monitored cell cycle distribution and proliferation. However, apart from a transient increase of the proportion of G1 cells after 24 hours (*P* = 0.02, Supplemental Figure 5B, C), no significant effects on proliferation or cell cycle distribution were observed upon SOX11 (Supplemental Figure 5D). Similarly, primary MCL cells with low SOX11 expression showed a 1.5-fold higher IC_50_ value compared to MCL cells with high SOX11 expression (Supplemental Figure 6A-C).

It should be noted that primary MCL cells do not proliferate in culture and given that ara-C targets DNA replication in cycling cells, the mode of action of ara-C in non-dividing cells could be different.

To gather further evidence that SOX11-mediated ara-C sensitization is dependent on SAMHD1, we addressed the effect of SOX11 overexpression with and without concomitant Vpx-mediated SAMHD1 depletion in the SOX11-inducible JVM-2 system. Reproducibly, SAMHD1 depletion in SOX11-negative JVM-2^vector^ cells reduced the IC_50_ of ara-C by a factor of ~15 (Figure 3E). Induction of SOX11 in JVM-2^iSOX11^ treated with control VLPs (dX) led to a ~10-fold reduction of the IC_50_ of ara-C (Figure 3E & Supplemental Figure 6D). The enhanced cytotoxicity of ara-C treatment following Vpx-mediated SAMHD1 depletion in JVM-2^iSOX11^ or JVM-2^vector^ was recapitulated by increased DNA damage responses as evidenced by increased levels of cleaved Poly [ADP-ribose] polymerase 1 (PARP1), phosphorylated checkpoint kinase 2 (p-ChK2), cleaved caspase-3 and γ-H2A.X as compared to their control VLP-treated counterparts (Figure 3F). Similar results were observed for ara-C treatment of SOX11-induced JVM-2^iSOX11^ without SAMHD1 depletion, albeit to a lesser extent, which might be explained by an incomplete induction of SOX11. Consistent with this notion, SOX11 was induced in ~40% of JVM-2^iSOX11^ cells upon treatment with 0.1μM doxycycline (Supplemental Figure 4 C-E) whereas Vpx treatment led to complete ablation of SAMHD1 protein. SAMHD1 depletion equally sensitized JVM2^vector^ and JVM-2^iSOX11^ to ara-C, indicating that SOX11-mediated ara-C sensitization is SAMHD1-dependent (Figure 3E & Supplemental Figure 6D). In line with this, the effect of ara-C on apoptotic and DNA damage markers in SAMHD1 depleted JVM-2^vector^ and JVM-2^iSOX11^ cells were very similar (Figure 3F). Taken together, these results indicated that SOX11-mediated reduction of tetrameric SAMHD1 translates into inhibition of SAMHD1 ara-CTPase activity.

Since ara-C efficacy is directly correlated to intracellular accumulation of ara-CTP that can be reduced by SAMHD1 ara-CTPase activity^14^, we hypothesized that induction of SOX11 in JVM-2^iSOX11^ would lead to an increase in ara-CTP. As predicted, ara-CTP levels increased when SOX11 was induced (Figure 3G). Since levels of dNTPs were lower in JVM2^iSOX11^ as compared to vector cells (Supplemental Figure 7B, C), we normalized ara-CTP levels to dNTPs, resulting in significant increase of ara-CTP-to-dTTP ratios by a factor of 1.5 (*P* = 0.0076) in the presence of SOX11 as compared to SOX11-negative vector control cells following 24 hours treatment with ara-C (Figure 3G and Supplemental Figure 7A).

### SOX11 enhances ara-C sensitivity of MCL to ara-C *in vivo*

**Figure 4:**
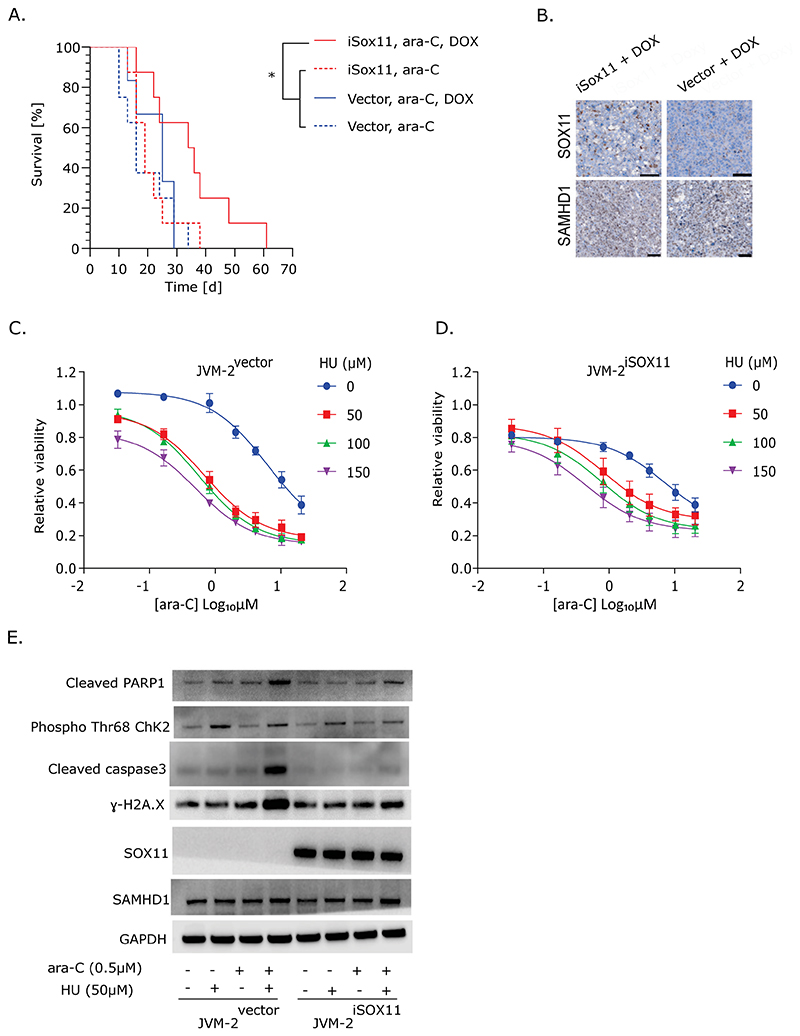
SOX11 sensitizes MCL to ara-C treatment *in vivo* and HU mimics SOX11-mediated sensitization to ara-C in SOX11-negative MCL cell lines. A. Kaplan–Meier analysis of NOD/SCID mice injected with JVM-2^vector^ or JVM-2^iSOX11^ that received ara-C (100 mg/kg) after 5 days of injection with cells versus untreated controls. n = 8 per group. B. Immunohistochemistry revealing SOX11 and SAMHD1 staining in formalin-fixed paraffin-embedded (FFPE) tumor tissue from mice injected with either JVM-2^vector^ or JVM-2^iSOX11^ treated with doxycycline. Scale bar = 50 μm. C & D. Dose response curves for cytarabine when combined to the indicated concentrations of HU (on the right side of the curves) in JVM-2^vector^ and JVM-2^iSOX11^, respectively. Cells were treated with 0.1 μM doxycycline for 24 h before combined treatment with ara-C and HU which lasted for 72 h until viability was measured using CellTiter-Glo® Luminescent Cell Viability Assay. Data of three independent experiments are represented as mean ± SEM. E. Western blot analysis of apoptosis and DNA damage markers upon single treatment of ara-C (0.5 μM) or HU (50 μM) or their combination versus the respective untreated controls in JVM-2^vector^ and JVM-2^iSOX11^. After 24 h of 0.1 μM doxycycline treatment, combined or single treatments were performed for 24 h. The blot is a representative out of three independent biological replicates. Protein sizes: cleaved PARP1 (89 KDa), SOX11 (74 KDa), SAMHD1 (71 KDa), phospho-ChK2 (T68) (~62 KDa), GAPDH (37 KDa), cleaved caspase3 (17 KDa) and γ-H2A.X (14 KDa).

To validate the relevance of SOX11-mediated SAMHD1 inhibition *in vivo*, we xenotransplanted JVM-2^iSOX11^ or JVM-2^vector^ cells subcutaneously into NMRI nude mice, supplementing doxycycline in the drinking water to induce SOX11 expression (Supplemental Figure 8A). When the subcutaneous tumors reached the threshold volume of 150 mm^3^ the mice received once daily intraperitoneal injections of 100 mg/kg ara-C i.p. for 5 consecutive days. Median survival time was significantly prolonged in mice with JVM-2^iSOX11^ tumors supplemented with doxycycline and treated with ara-C (35 days) as compared to their counterparts without doxycycline (19 days) (Figure 4A, *P* = 0.04). The increase of survival was dependent on the presence of inducible *SOX11*, as doxycycline-supplemented mice with JVM-2^vector^ tumors treated with ara-C had a significantly shorter median survival (24 days) (Figure 4A, *P* = 0.04). These results are mirrored in the tumor volumes, which were significantly lower in JVM-2^iSOX11^ mice with doxycycline treated with ara-C versus either JVM-2^iSOX11^ mice without doxycycline with ara-C (Supplemental Figure 8B, *P* < 0.0001) or JVM-2^vector^ with doxycycline and ara-C (Supplemental Figure 8C, *P* = 0.01). Posthumous immunohistochemical staining of tumors confirmed the presence of SOX11 in doxycycline-treated JVM-2^iSOX11^ tumors, but not in JVM-2^vector^, whereas SAMHD1 was expressed under all conditions (Figure 4B).

### Hydroxyurea sensitizes SOX11-negative MCL to ara-C *in vitro*

We have previously reported that non-competitive inhibitors of ribonucleotide reductase (RNR) including hydroxyurea (HU) inhibit SAMHD1 ara-CTPase activity, thereby potentiating sensitivity to ara-C in AML^36, 42^. Given the inhibitory effects of SOX11 on SAMHD1 ara-CTPase, we hypothesized that SOX11-negative MCL might disproportionately benefit from the recently identified pharmacological inhibitors of SAMHD1 as compared to SOX11 positive MCL cells^36^. To test this, we treated JVM2^vector^ and JVM-2^iSOX11^ cells with increasing concentrations of ara-C and HU. HU reduced the IC_50_ of ara-C in JVM-2^vector^ in a dose-dependent manner by up to 15-fold (Figure 4C). The sensitizing effect of HU was less pronounced in JVM-2^iSOX11^ (Figure 4D), in which 40% of the cells express SOX11 (Supplemental Figure 4D). Drug-drug interaction analyses calculating Zero Interaction Potencies (ZIP) confirmed a substantially higher synergy of HU and ara-C in JVM-2^vector^ than JVM-2^iSOX11^ (Supplemental Figure 9A, B). Addition of HU to ara-C as compared to ara-C alone led to a more pronounced increase in DNA damage responses in JVM-2^vector^ as compared to JVM-2^iSOX11^(Supplemental Figure 9C-F). The reduced synergy of HU/ara-C combinations in the presence of SOX11 were consistent with SOX11-mediated SAMHD1 inhibition (Figure 4E).

### Regulation of SAMHD1 expression in MCL

We have previously shown that there is a weak positive correlation of SOX11 and SAMHD1 expression based on immunohistochemistry (N=62, Spearman correlation coefficient 0.27, *P* = 0.036). ^19^ To further investigate this, we analyzed gene expression data of 44 previously published MCL cases ^43^ and confirmed a positive correlation of SAMHD1 and SOX11 expression (Spearman rank correlation R=0.37; *P*=0.013) (Supplemental Figure 10A). However, the correlation was mainly driven by lower SAMHD1 expression in non-nodal MCL (nnMCL) (Supplemental Figure 10C) and lost when restricting the analysis to conventional MCL (cMCL) (Supplemental Figure 10B). Consistently, silencing of SOX11 in SOX11-positive MCL cell lines ^19^ or induction of SOX11 in SOX11-negative JVM-2 (Figure 3F, Supplemental Figure 10D) did not affect SAMHD1 expression. Analyses of SAMHD1 histone marks and DNA methylation together with SAMHD1 mRNA expression in 5 published MCL cases (2 cMCL and 3 nnMCL) in comparison with normal B-cells ^43, 44^ did furthermore not reveal evidence of epigenetic regulation of differential SAMHD1 expression in MCL (Supplemental Figure 10E). Looking at the expression of SAMHD1 measured by RNAseq in these 5 MCL cases, 2 cMCL and 2 nnMCL showed similar levels, and one nnMCL displayed lower expression (Supplemental Figure 10F). However, all 5 cases showed a similar epigenomic profile and therefore, SAMHD1 expression differences in MCL could be a consequence of other changes not related to epigenetic regulation.

## Discussion

Throughout the past decade, understanding of MCL pathobiology has witnessed remarkable progress, with respect to both intrinsic cellular anomalies and tumor microenvironment.^4, 45^ This has led to promising therapeutic approaches to overcome refractoriness to therapy and relapse in MCL, including the use of non-covalent BTK inhibitors, immunomodulatory agents, bispecific antibodies, and next generation cell-based therapies.^3, 45^ However, rituximab-based immunochemotherapy remains the backbone strategy for MCL treatment.^5, 6, 46^ The Nordic regimen R-CHOP (cyclophosphamide, doxorubicin, vincristine, and prednisone + rituximab) coupled to high-dose ara-C improved MCL outcomes.^6, 7^ High-dose ara-C has been shown to effectively prolong time to treatment failure in MCL patients who received R-CHOP followed by ASCT.^5, 9^ It is therefore incumbent to adequately understand of the regulatory underpinnings of ara-C response in MCL.

Here, we provide a novel mechanistic insight into how ara-C response is regulated by SAMHD1 in MCL. Unbiased co-immunoprecipitation of SOX11 revealed SAMHD1 as the top significant binding-partner of SOX11 in MCL cell lines. The SOX11-SAMHD1 interaction was validated by proximity-ligation assays, and cellular thermal shift assays. Direct binding of the SOX11 HMG domain with SAMHD1 was demonstrated by Microscale Thermophoresis. *In situ* crosslinking revealed that SOX11 binding to SAMHD1 reduced SAMHD1 tetramerization. Consequently, this interaction triggered inhibition of ara-CTPase activity, leading to higher intracellular ara-CTP accumulation and sensitization of *in vitro* and *in vivo* MCL models to ara-C treatment. *In vitro* studies also showed that pharmacological inhibition of SAMHD1 by HU can mimic the ara-C-sensitizing effect of SOX11 in SOX11-negative MCL. Altogether, these findings substantiate the negative regulatory effect of SOX11 on SAMHD1’s ara-CTPase activity through physical binding, without affecting SAMHD1 gene expression. Recently, we demonstrated that SAMHD1 expression levels show no association to survival in MCL patients receiving ara-C^19^. In line with this, Roider *et al* showed that SAMHD1 expression and mutation status did not correlate with failure-free survival or complete remission rate in MCL patients who received ara-C treatment^18^. As most cases of MCL included in clinical studies are of the conventional subtype and thus SOX11-positive^21, 22^ (in contrast to the more indolent, non-nodal leukemic MCL variant which is SOX11 negative^23^), it can be inferred that SAMHD1 is inherently inhibited by SOX11. Our *ex vivo* functional studies showed that primary MCL with low expression of SOX11 showed higher IC_50_ in response to ara-C than primary cells derived from SOX11-high MCL cases. The present work appears to be able to explain the lack of correlation between SAMHD1 levels and ara-C efficacy by intrinsic SOX11-mediated inhibition of SAMHD1 in MCL. Consistently, in a study from the European MCL Network with patients treated with ara-C containing regimens, cases with low SOX11 expression (<10% of SOX11+ tumor cells) had a shorter time-to-treatment failure and shorter overall survival as compared to SOX11 positive cases^8^. Similar results have been reported in the Nordic MCL 2/3 cohort.^47^ This could be ascribed to the lack of SOX11-mediated sensitization to ara-C as indicated by our findings. It could also be suggested that HU could be a promising strategy to increase ara-C sensitivity in SOX11-negative or -low MCL as recently shown for AML^17^. However, it should be noted that other features of SOX11 negative MCL, such as frequent *TP53* aberrations^8, 48, 49^ might also contribute to different outcomes.

Two previous studies reported a SAMHD1 mutation rate in MCL of ~8%, but no significant correlation with SAMHD1 gene expression could be identified^18, 50^ Whether these mutations affect the binding to SOX11 or the response to ara-C in MCL is currently not known. Further biochemical studies are needed to define the SAMHD1-SOX11 binding interface more precisely and to explore whether SOX11 modulates other functions of SAMHD1 and, in extension, the pathobiology of MCL.

We conclude that SAMHD1 ara-CTPase activity is intrinsically inhibited by SOX11 in MCL which could explain the efficacy of ara-C containing regimens in younger and elderly patients with MCL^10^. It is therefore tempting to speculate that SOX11 expression level could be used to stratify MCL treatment. It is also appealing to investigate SOX11 and SAMHD1 expression in parallel to define cut-off levels of SOX11 sufficient to overcome SAMHD1-mediated ara-C resistance at clinically relevant doses.

## Supplementary Material

Supplemental Table 1

Supplementary Information

## Data Availability

The MS data has been deposited in the ProteomeXchange database under the accession code PXD030976. During review the data can be accessed via: URL: https://repository.jpostdb.org/preview/2072219057649edcd204bc8,, Access key: 9780.

## Key Points

- SOX11 directly binds, via its HMG domain, to SAMHD1, reducing its tetramerization and inhibiting its ara-CTPase activity in MCL.
- The non-competitive inhibitor of SAMHD1, hydroxyurea, sensitizes SOX11-negative MCL to ara-C.
