# Supplementary material for: SOX11 is a novel binding partner and endogenous inhibitor of SAMHD1 ara-CTPase activity in mantle cell lymphoma
Source: Blood. Author manuscript; Available in PMC 2024 May 21. (PMC11103171; doi:10.1182/blood.2023022241)
Supplement: Supplementary Information [file EMS193709-supplement-Supplementary_Information.pdf]

Supplemental Table 1

Information on primary MCL samples.

Sample	Diagnostic sample	Cell sample site	Patient gender/age	SOX11 by IHC*	Lymphoma cells
PS1	yes	Lymph node	M/83	Positive (79%)	91%
PS2	yes	Lymph node	M/71	Positive (93%)	95%
PS3	yes	Bone marrow	M/69	Low (15%)	91%
PS4	no	Lymph node	F/60	Low (12%)	55%
PS5	yes	Lymph node	F/69	Intermediate (48%)	69%

*as assessed by immunohistochemistry on the original tissue material from lymph node or bone marrow.

Supplemental Table 2

List of antibodies used in the study.

Antibody	Catalog number and company	Application and optimal dilution
Phospho-Chk2 (Thr68) Antibody #2661	2661T, Cell Signaling	Western blot (1:1000)
SOX-11 (MRQ-58) Mouse Monoclonal Antibody	382M-14, Cell Marque	Immunofluorescence (1:50)
Anti-SOX11 antibody [EPR8192]	ab134107, Abcam	Western blot (1:1000) Immunofluorescence (1:100)
Phospho-Histone H2A.X (Ser139) Monoclonal Antibody	MA1-2022, ThermoFisher	Western blot (1:1000)
Rabbit IgG HRP Linked Whole Ab Cytiva NA934	GENA934-1ML, Merck	Western blot (1:2000)
Cleaved PARP (Asp214) Antibody (Human Specific) #9541	9541S, Cell signaling	Western blot (1:1000)
Cleaved Caspase-3 (Asp175) (5A1E) Rabbit mAb #9664	9664S, Cell signaling	Western blot (1:1000)
SAMHD1 Antibody, A303-691A	A303-691A, Bethyl	Western blot (1:1000) Immunofluorescence (1:200)
Anti-SAMHD1 antibody [OTI1A1], 100 ul	ab128107, Abcam	Western blot (1:1000) Immunofluorescence (1:100)
Phospho-SAMHD1 (Thr592) (D7O2M) Rabbit mAb #89930	89930S, Cell signaling	Western blot (1:1000)
GAPDH (14C10) Rabbit mAb	2118S, Cell signaling	Western blot (1:1000)
Anti-SOD-1 (SOD-1 (G-11)	SC-17767, Santa cruz	Western blot for CETSA (1:2000)
ECL peroxidase labelled anti-mouse IgG antibody	NA931VS, GE Healthcare	Western blot (1:2000)

Supplemental Figure 1

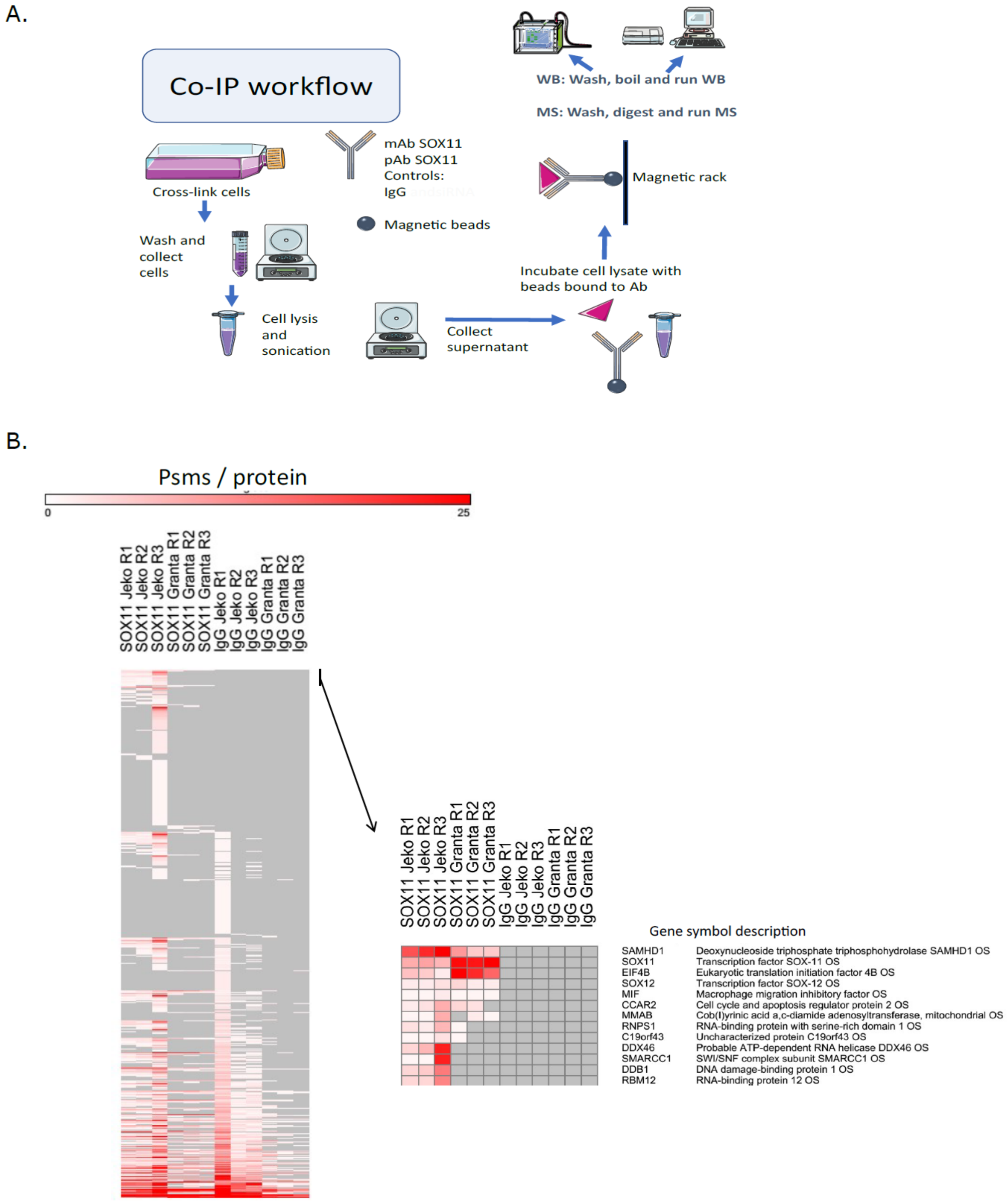

Supplemental Figure 1 | Identification of SOX11-binding proteins in MCL cell lines by Co-immunoprecipitation/mass spectrometry

A. Graphical illustration of workflow of co-immunoprecipitation and sample preparation for mass spectrometry.

B. Heatmap of mass spectrometric analysis shows the top significant binding proteins of SOX11 compared to IgG in Granta-519 and JeKo-1 (n=3). Proteins are sorted according to PSM values (20 for Granta-519 & 85 for JeKo-1) and SAMHD1 is ranked as the highest significant partner protein. On the top, a scale of PSM values (0-25). This figure is related to Figure 1A (in the main manuscript).

Supplemental Figure 2

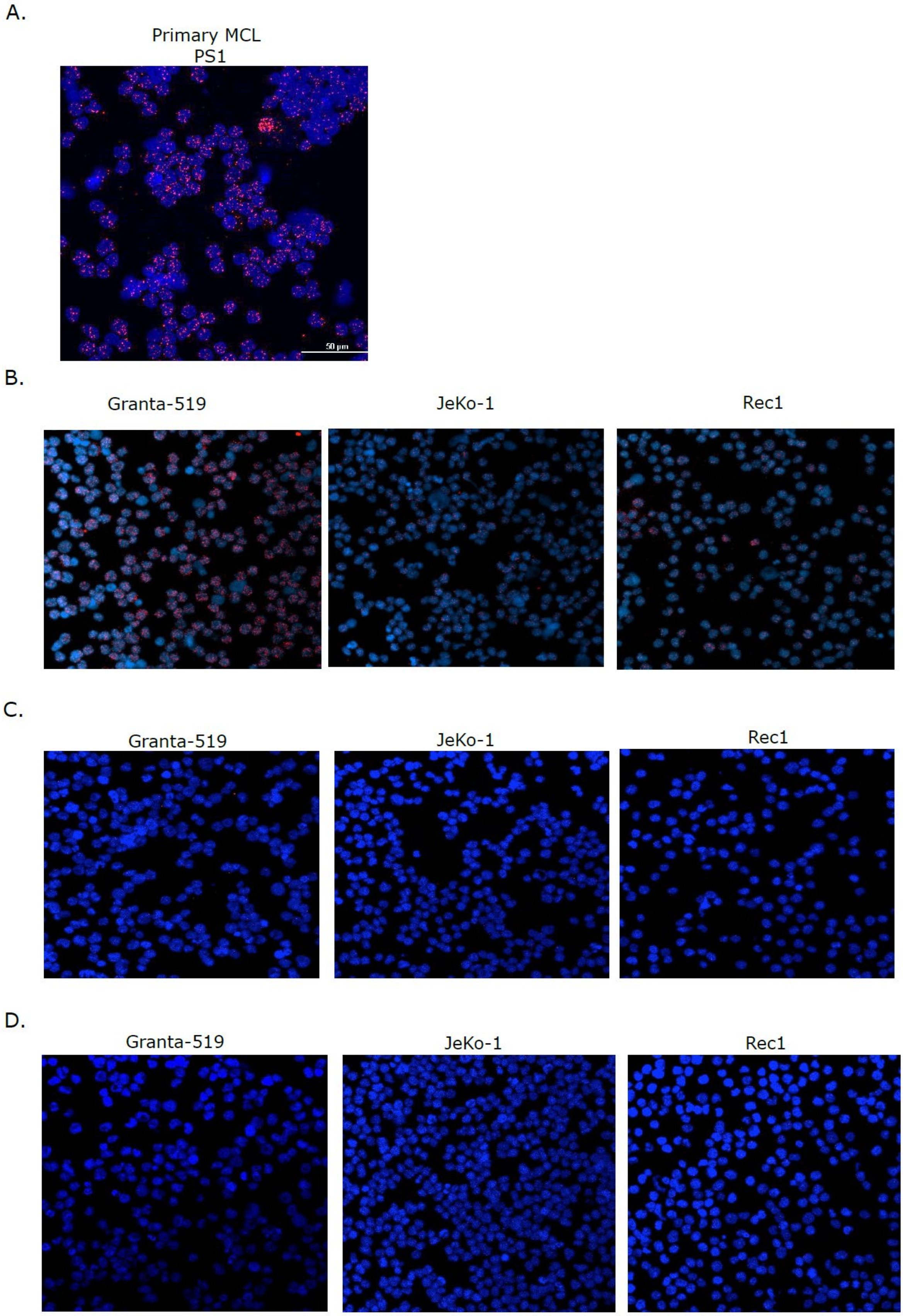

Supplemental Figure 2 │ SOX11-SAMHD1 co-localization in primary MCL and MCL cell lines by PLA

Proximity ligation assay performed on A. Primary MCL (PS1), B. Granta-519, JeKo-1 and Rec1 cells using rabbit polyclonal anti-SAMHD1 (Bethyl) and mouse monoclonal anti-SOX11 (MRQ-58). DAPI channel represents stained nuclei, whereas red channel (TRITC) represents SOX11-SAMHD1 colocalization. Magnification 60x, scale bar 50 μm and pinhole set at 1.2. The figure is related to Figure 1B (in the main manuscript).

PLA performed on Granta-519, JeKo-1 and Rec1 using single antibody against SOX11 (C) or against SAMHD1 (D), respectively, as negative control for the PLA. The figures are related to Figure 1C (in the main manuscript).

Supplemental Figure 3

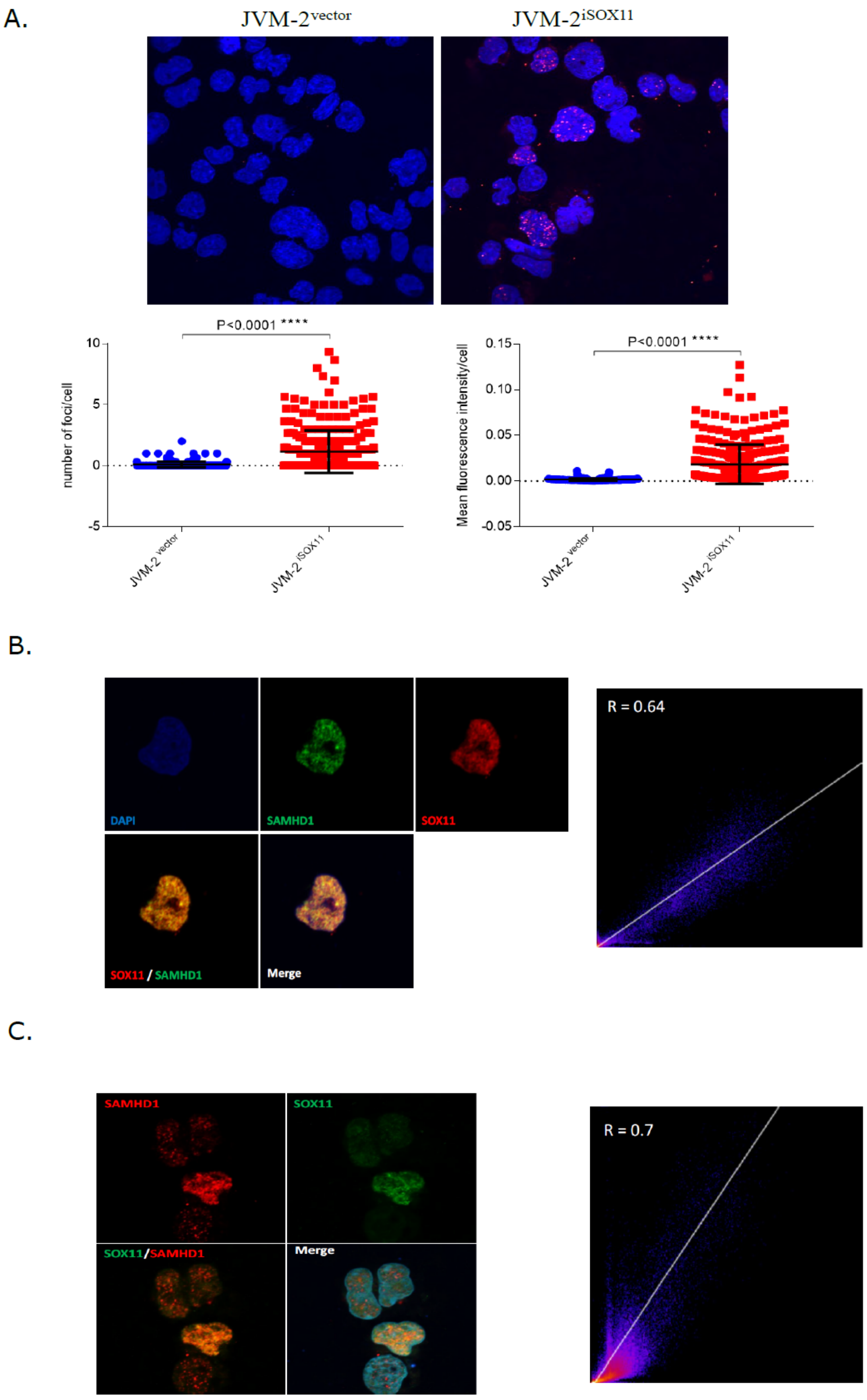

Supplemental Figure 3│Ectopically expressed SOX11 co-localizes with endogenous SAMHD1 in inducible JVM-2

A. Top, representative pictures of proximity ligation assay performed on JVM-2^{vector} and JVM-2^{iSOX11} using mouse monoclonal anti-SOX11 (MRQ-58) and rabbit polyclonal anti-SAMHD1 (Bethyl). Bottom, number of foci per cell and mean fluorescence intensity per cell. Magnification 60x and pinhole is set at 1.2. This figure is related to Figure 1D-F (in the main manuscript).

B. Immunofluorescence confocal imaging of JVM-2^{iSOX11} using mouse monoclonal anti-SAMHD1 (Abcam) and rabbit polyclonal anti-SOX11 (Abcam). To the right, Pearson correlation indicates the colocalization of the aligned staining channels for both proteins. This correlation was performed using ImageJ software. Magnification 100x and pinhole is set at 1.2. The figure is related to Figure 1D-F (in the main manuscript).

C. Immunofluorescence confocal imaging of JVM-2^{iSOX11} using mouse monoclonal anti-SOX11 (MRQ-58) and rabbit polyclonal anti-SAMHD1 (Bethyl). This figure is related to Figure 1D-F (in the main manuscript).

Supplemental Figure 4

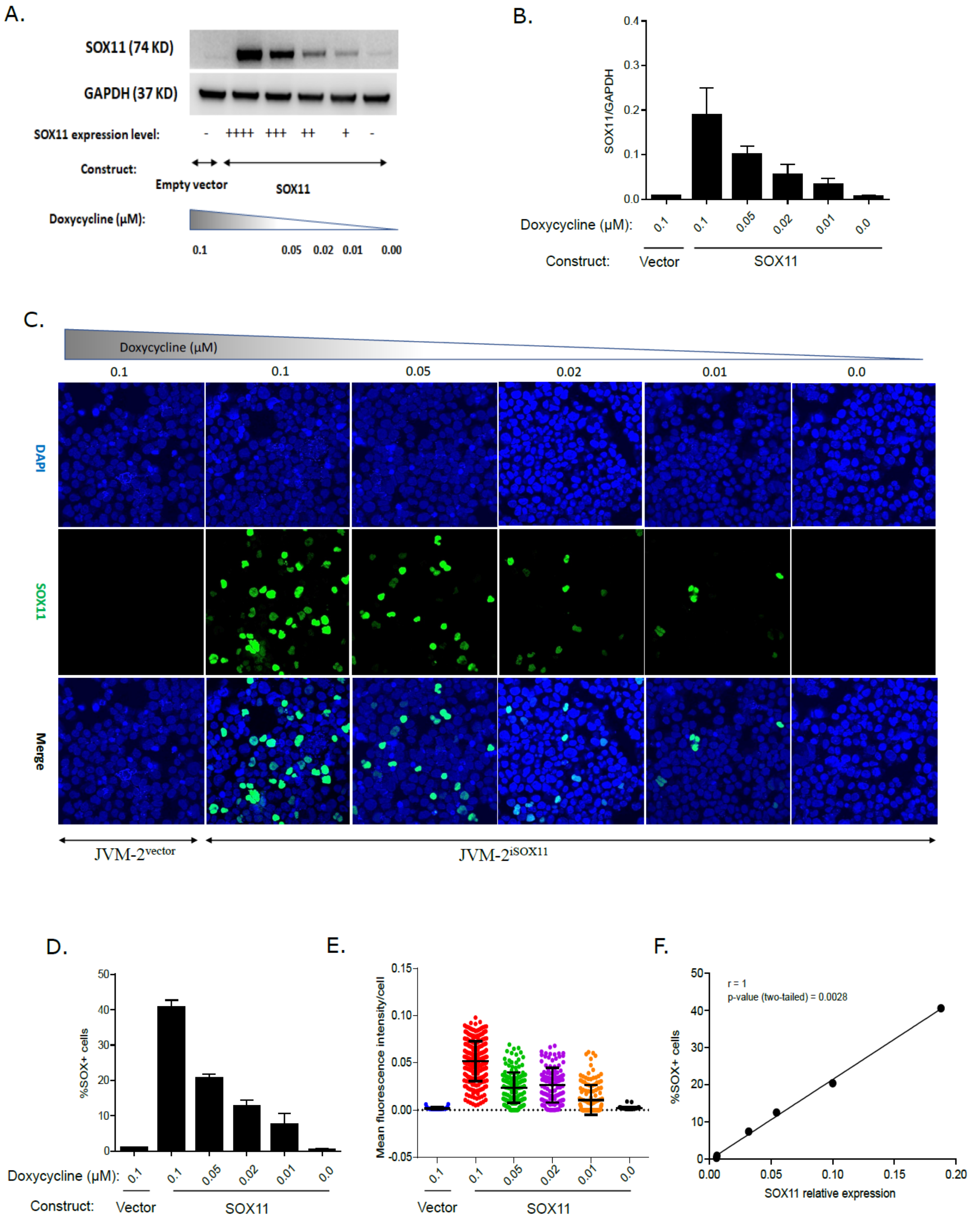

Supplemental Figure 4 | Doxycycline-induced overexpression of SOX11 in JVM-2

A. Representative western blot, out of three repeats, shows SOX11 expression in JVM-2^{vector} and JVM-2^{iSOX11} cultured in media containing the indicated concentrations of doxycycline for 96 h.

B. Relative expression of SOX11 calculated as normalized band intensity to GAPDH from western blot performed on JVM-2^{vector} and JVM-2^{iSOX11} cultured in media containing the indicated concentrations of doxycycline for 96 h (related to A). Data are represented as mean ± SEM of three independent experiments.

C. Immunofluorescence demonstrates the expression of SOX11 in JVM-2^{vector} and JVM-2^{iSOX11} cultured in media containing the indicated concentrations of doxycycline for 96 h. Magnification was set at 60x and pinhole at 1.2. DAPI channel represents stained nuclei, whereas green channel (FITC) represents SOX11 staining. One representative experiment out of three is shown. Cells were collected from the same experiment but were divided into two parts for the downstream processing for western blot and for immunofluorescent staining.

D. Percentage of SOX11-positive cells in total 900 cells of JVM-2^{vector} and JVM-2^{iSOX11} cultured in media containing the indicated concentrations of doxycycline. Related to C. Data are represented as mean ± SEM of three independent biological repeats.

E. Mean fluorescence intensity per cell in total 900 cells of JVM-2^{vector} and JVM-2^{iSOX11} cultured in media containing the indicated concentrations of doxycycline. Data are represented as mean ± SEM of three independent biological replicates.

F. Spearman rank correlation of average SOX11 relative expression (from B) and average percentage of SOX11-positive cells (from D). Both r and *P* (two-tailed) are indicated on the plot.

Supplemental Figure 5

A.

	Doxycycline (µM)	JVM-2^{iSOX11} 0.1	0.05	0.02	0.01	0.0
JVM-2^{vector}	0.1	<0.0001 ****	<0.0001 ****	0.0002 ***	0.013 *	0.73 ns
JVM-2^{iSOX11}	0.1		0.001 **	<0.0001 ****	<0.0001 ****	<0.0001 ****
	0.05			0.16 ns	0.004 **	<0.0001 ****
	0.02				0.17 ns	0.0018 **
	0.01					0.051 ns
	0.00					

Two-way ANOVA
****, p-value (two-tailed) < 0.0001
***, p-value (two-tailed) < 0.001
**, p-value (two-tailed) < 0.01
*, p-value (two-tailed) < 0.05
ns, p-value (two-tailed) ≥ 0.05

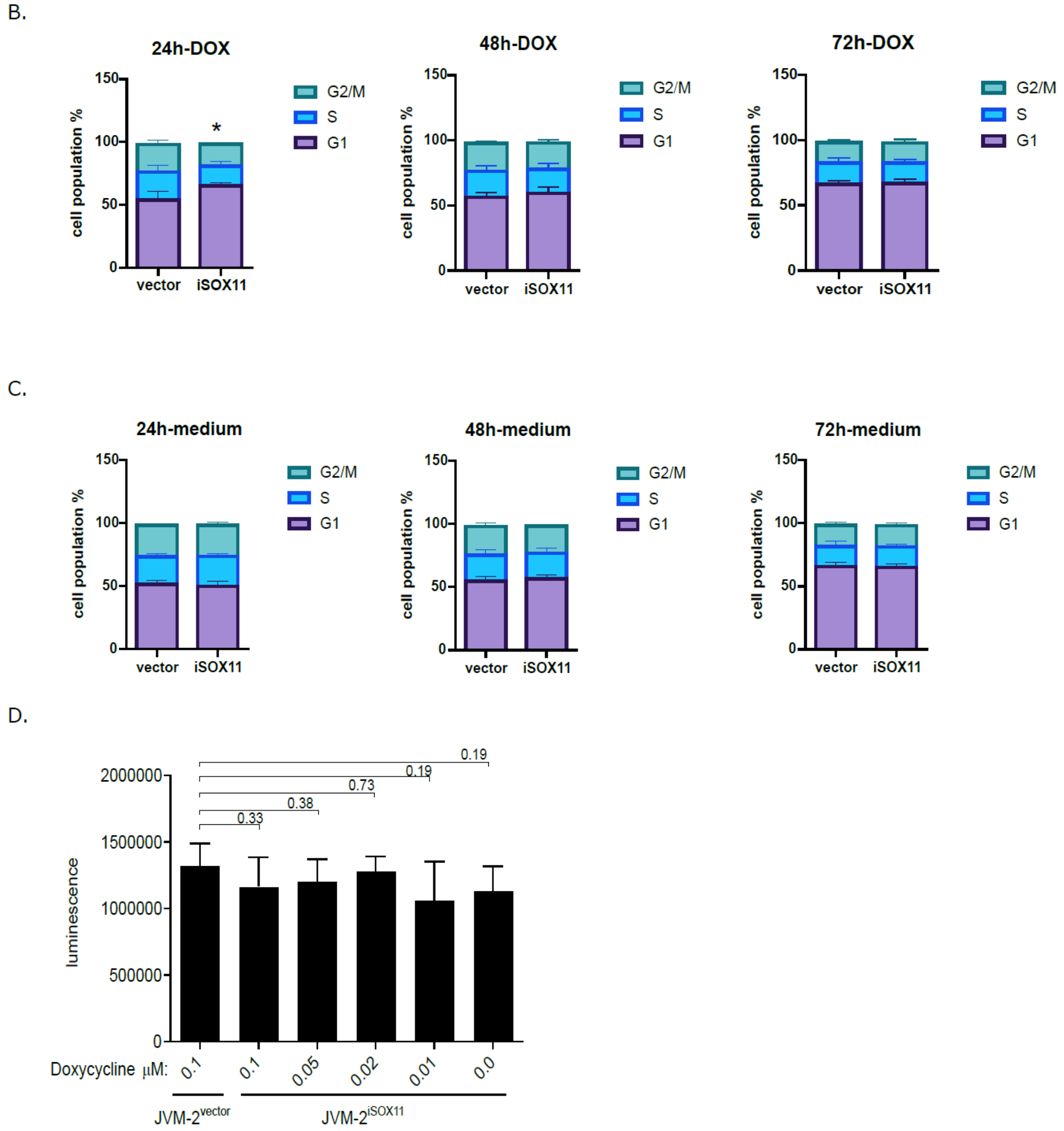

Supplemental Figure 5 | Effect of SOX11 induction in JVM-2 on viability and cell cycle

A. Matrix showing *P* (two-way ANOVA) between the different conditions of doxycycline concentrations. This figure is related to Figure 3D (in the main manuscript).

B. Cell cycle analysis of JVM-2^{vector} and JVM-2^{iSOX11} at 24, 48 and 72 hours following treatment with 0.1 μM doxycycline. The number of cells in different cell cycle stages (G1, S, G2/M) are presented as percentage (%) of the selected cell population, with mean ± SEM of four independent biological repeats. An unpaired t-test was used to determine statistical differences between the amount of JVM-2^{vector} and JVM-2^{iSOX11} cells in the different cell cycle stages; **P* (Unpaired t test, two-tailed) <0.05.

C. Cell cycle analysis of JVM-2^{vector} and JVM-2^{iSOX11} at 24, 48 and 72 hours in cells not treated with doxycycline. The number of cells in different cell cycle stages (G1, S, G2/M) are presented as percentage (%) of the selected cell population, with mean ± SEM of four independent biological repeats. An unpaired t-test was used to determine statistical differences between the amount of JVM-2^{vector} and JVM-2^{iSOX11} cells in the different cell cycle stages.

D. Cell viability after 96 h of induction of SOX11 expression with 0.1 μM doxycycline. Luminescence values of JVM-2^{vector} and JVM-2^{iSOX11} cultured in different concentrations of doxycycline. The data are represented as mean ± SEM for four independent biological replicates. The *P* indicated on the chart are calculated by performing unpaired, two-tailed t-test with Welch correction. This figure is related to Figure 3D (in the main manuscript).

Supplemental Figure 6

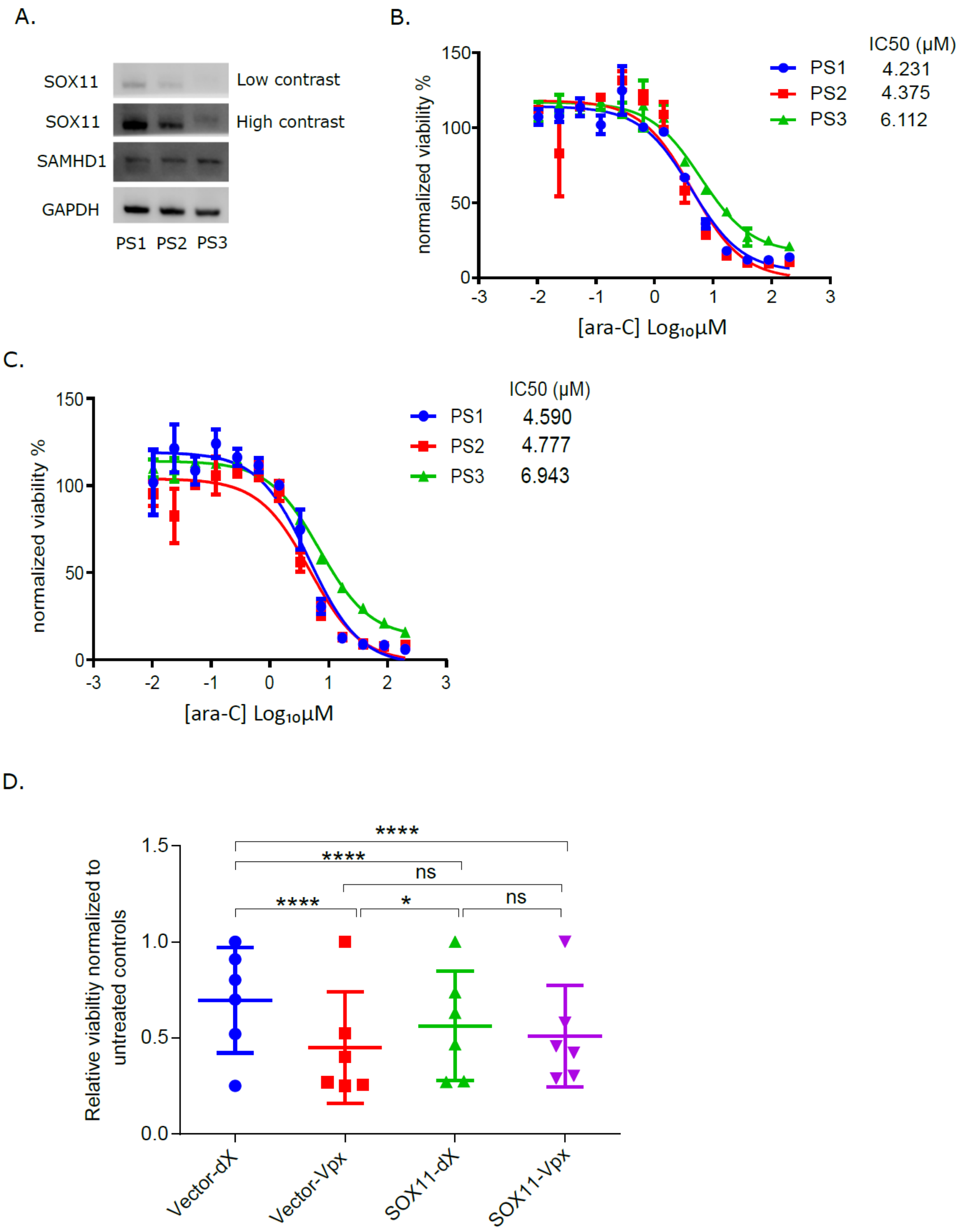

Supplemental Figure 6│Sensitivity to ara-C in primary MCL samples with different SOX11 expression and in JVM2 upon SOX11 induction.

A. Western blot shows SOX11, SAMHD1 and GAPDH in three primary MCL samples PS1-3 with different SOX11 expression levels (See Supplemental Table 1 for details).

B, C. Dose-response curve for ara-C using PS1-3 primary MCL (two independent experiments). The cells were treated with ara-C for 72h and cell viability was analyzed by CellTiter Glo assay. The values on the y-axis represent the relative viability values which were calculated by normalizing absorbance value at each dose of ara-C for each condition to respective untreated controls (normalized as 100%), whereas treatment with high-dose doxorubicin was set as the 0%.

D. Scatter plot of relative viability data from Figure 3E (in the main manuscript). P (two-tailed) was calculated by Two-way ANOVA analysis to compare different conditions: ****$P < 0.0001$, ***$P < 0.001$, **$P < 0.01$, *$P < 0.05$, ns $P \geq 0.05$.

Supplemental Figure 7

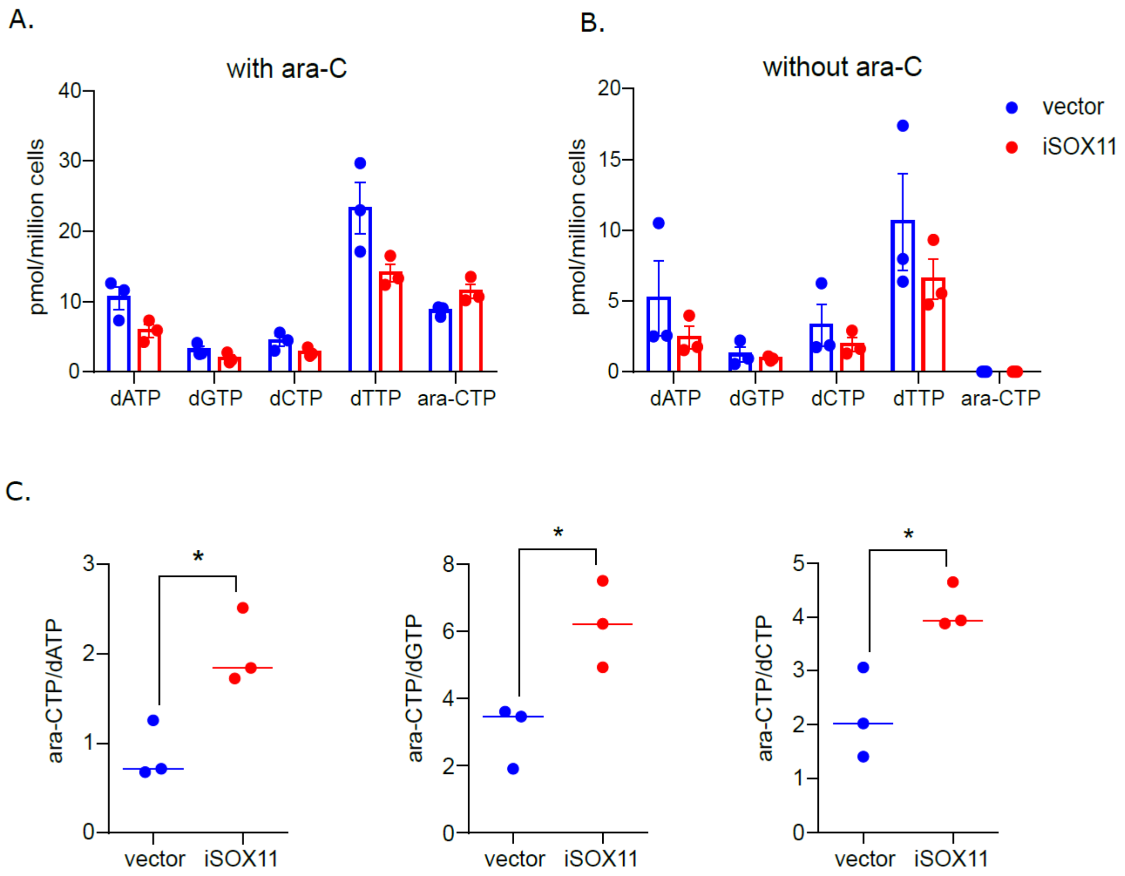

Supplemental Figure 7 | *SOX11* induction reduces intracellular level of ara-CTP in JVM-2

A-B. Absolute intracellular levels of dNTPs and ara-CTP in pmol/million cells in JVM-2^{vector} or JVM-2^{iSOX11} cells without (A) and with (B) ara-C (10 μM), after 24 h. Related to Figure 3G (in the main manuscript).

C. Intracellular ara-CTP levels normalized to levels of canonical dATP (left), dGTP (middle) or dCTP (right), determined using HPLC-MS/MS. Both JVM-2^{vector} and JVM-2^{iSOX11} were treated with 10 μM of ara-C for 24 h. Circles and error bars correspond to individual values, mean ± SEM of at three independent experiments. Analyses were performed using unpaired two-tailed t-tests. *$P < 0.05$. This figure is related to Figure 3G (in the main manuscript).

Supplemental Figure 8

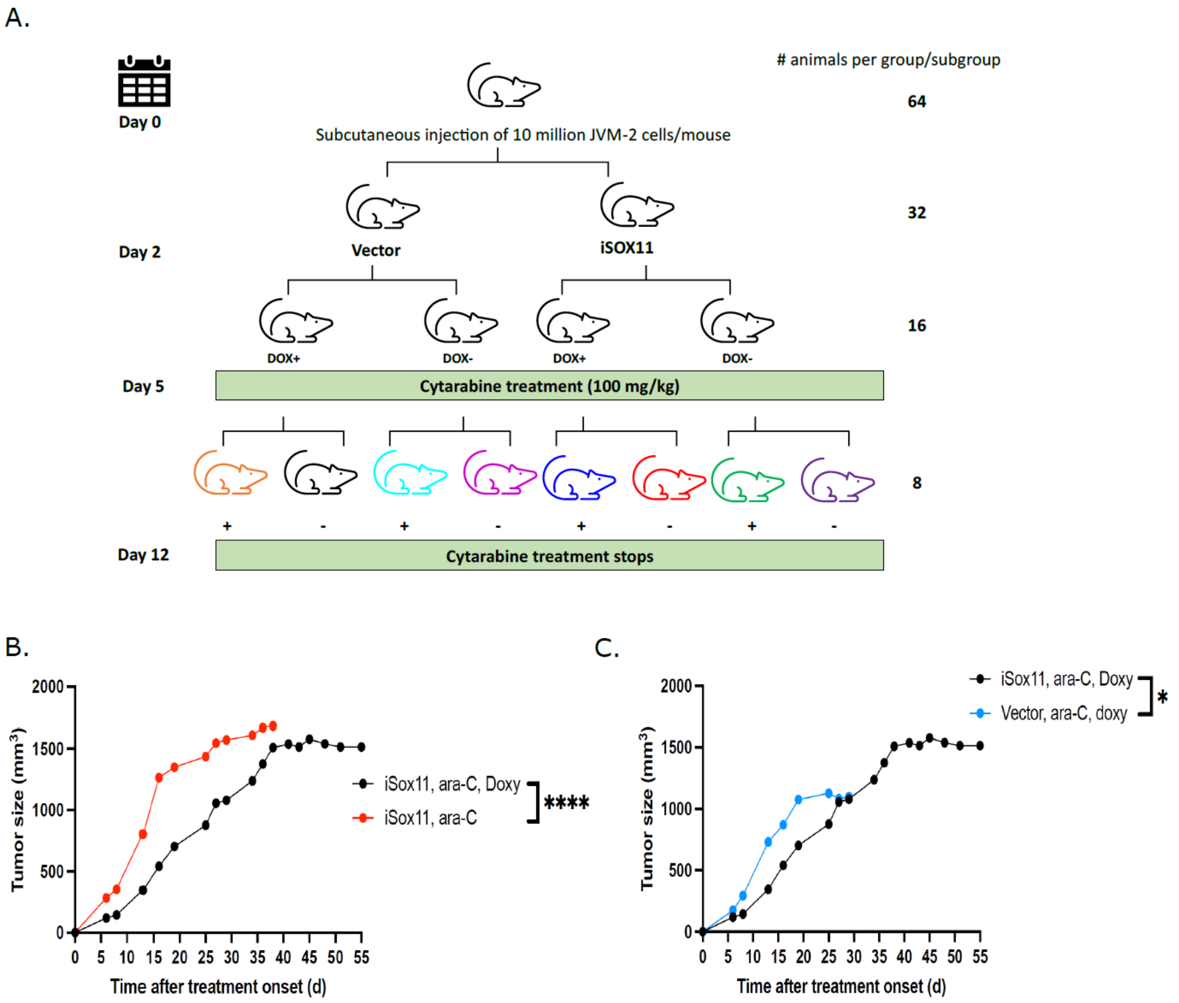

Supplemental Figure 8 │ *SOX11* induction in the presence of ara-C significantly reduced tumour sizes in mice injected with JVM-2 cells

A. Illustration of stratification of xenotransplated mice with JVM-2^{vector} or JVM-2^{iSOX11} and plan for ara-C and doxy treatment.

B. Average tumor volumes during and after ara-C treatment in mice injected with iSox11 cells treated with ara-C and doxycycline (black) and mice treated with ara-C alone (red). Lines represent average volumes for the groups. Statistical analysis was done using unpaired two-tailed t-test of the means. $t = 6.057$, df = 11, ****$P < 0.0001$.

C. Average tumor volumes during and after ara-C treatment in mice injected with iSox11 cells treated with ara-C and doxycycline (black) versus mice injected with vector cells treated with ara-C and doxycycline (blue). Lines represent average volumes for the groups. Statistical analysis was done using unpaired two-tailed t-test of the means. $t = 3.309$, df = 8, *$P < 0.05$.

Supplemental Figure 9

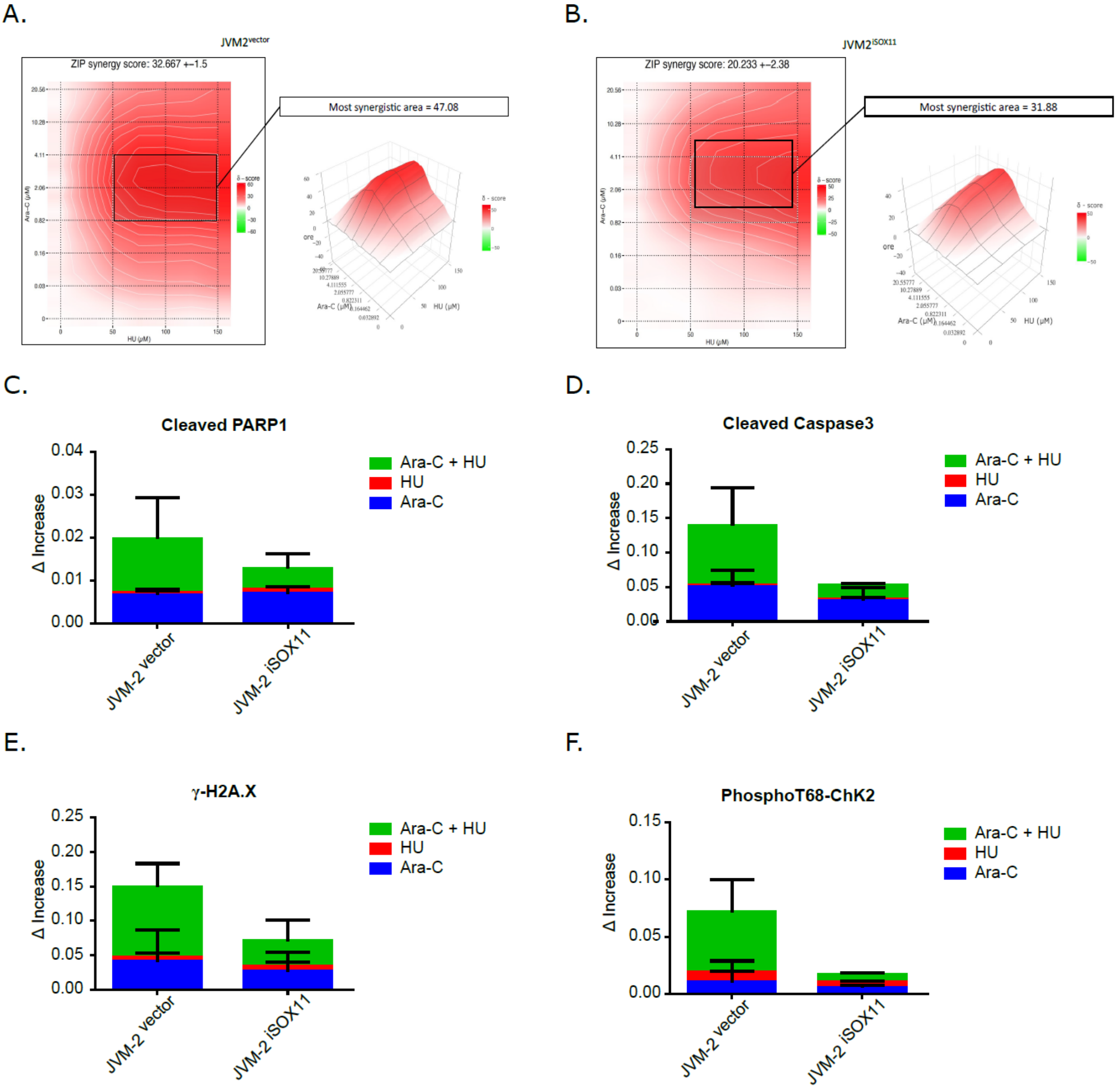

Supplemental Figure 9 | HU synergizes with ara-C in SOX11-negative JVM-2

A, B. Synergy analyses for the addition of HU to ara-C in JVM-2^{vector} (A) and JVM-2^{iSOX11} (B) cells using zero interaction potency, with heat maps showing areas of most synergy in red. Areas of highest synergy highlighted with concentrations of HU and ara-C on the x- and y-axis, respectively.

C-F. Semiquantitative analysis of Δ increase in the level of the indicated markers of apoptosis and DNA damage upon single treatment of HU or ara-C or their combination versus the respective untreated controls (related to Figure 4E in the main manuscript). The data are represented as mean ± SEM for three independent biological replicates.

Supplemental Figure 10

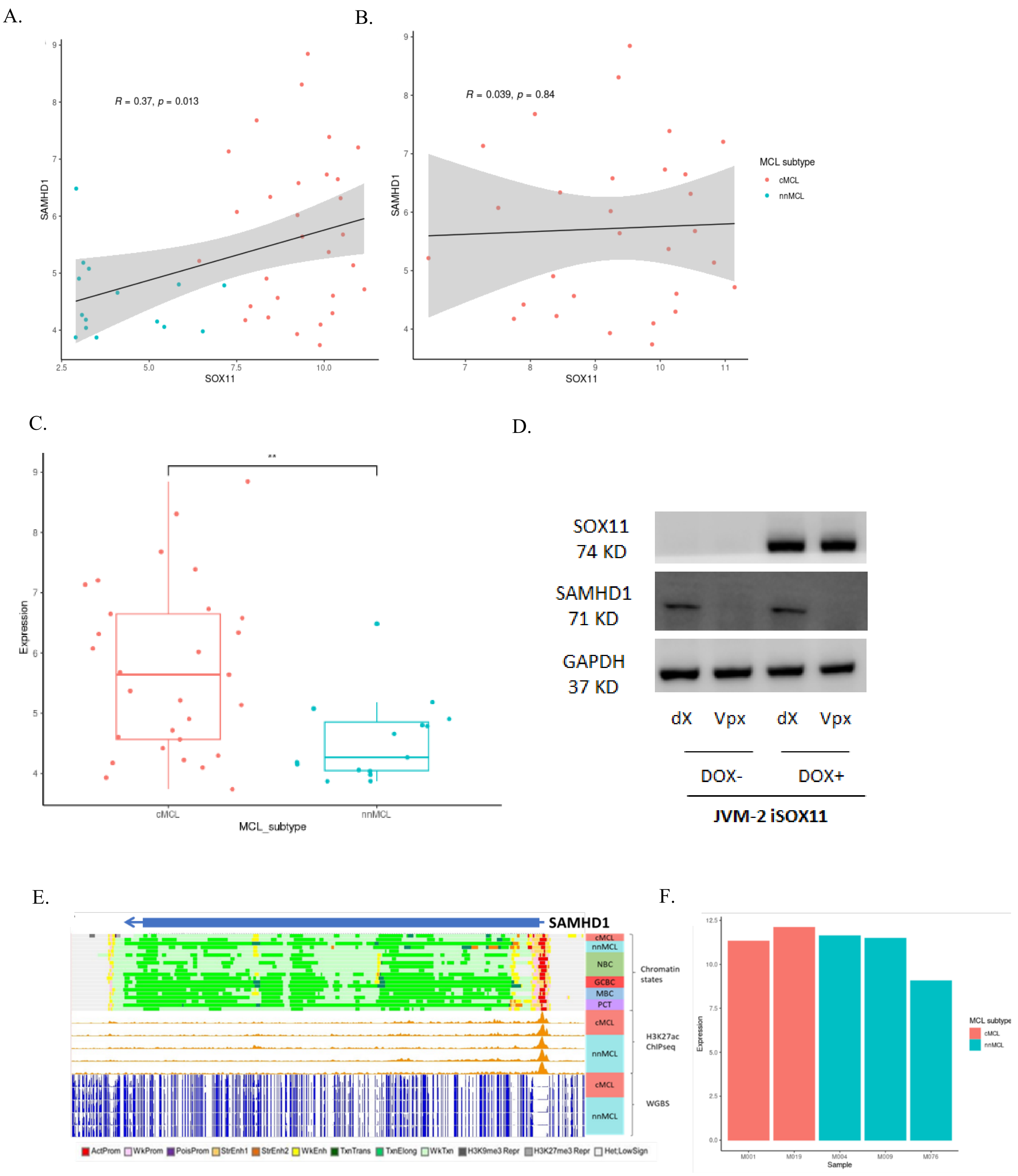

Supplemental Figure 10 │ Regulation of SAMHD1 expression in MCL.

A. Spearman correlation of expression levels of SOX11 and SAMHD1 in 44 MCL cases including 29 cMCL (in red) and 15 nnMCL (in blue).
B. Spearman correlation of expression levels of SOX11 and SAMHD1 in 29 cMCL cases.
C. Boxplot of expression of SAMHD1 in 44 MCL from RNA-sequencing (29 conventional (cMCL) cases and 15 leukemic non-nodal (nnMC) cases).
D. Western blot shows the expression of SOX11 (74 KD) and SAMHD1 (71 KD) in JVM-2^{iSOX11} in the indicated conditions. The experiment was performed to test the efficiency of doxycycline-mediated induction of SOX11 and SAMHD1 depletion by Vpx.
E. Representation of chromatin states including, H3K27ac ChIPseq peaks and methylation status analyzed by Whole-Genome Bisulfite Sequencing (WGBS) at SAMHD1 locus in 2 cMCL, 3 nnMCL cases and 15 normal B cells.
F. RNAseq expression data of SAMHD1 of the same MCL cases indicated in E (vst values).

Supplemental Figure 11

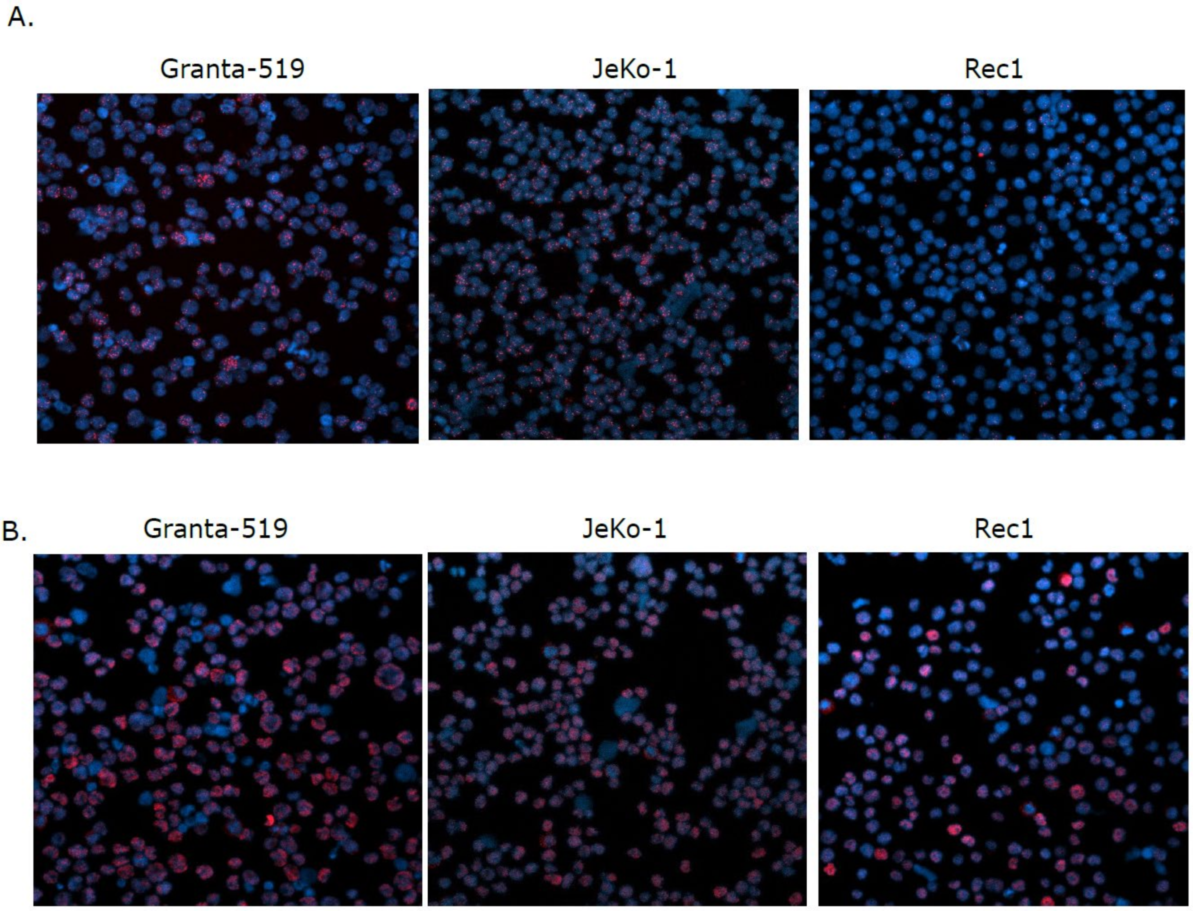

Supplemental Figure 11 | Testing the efficiency of PLA.

A, B. PLA performed on Granta-519, JeKo-1 and Rec1 using mouse and rabbit antibodies against SOX11 or against SAMHD1, respectively as positive control for the PLA. In A, two antibodies against SOX11 were used. In B, two antibodies against SAMHD1 were used.

Supplemental Figure 12

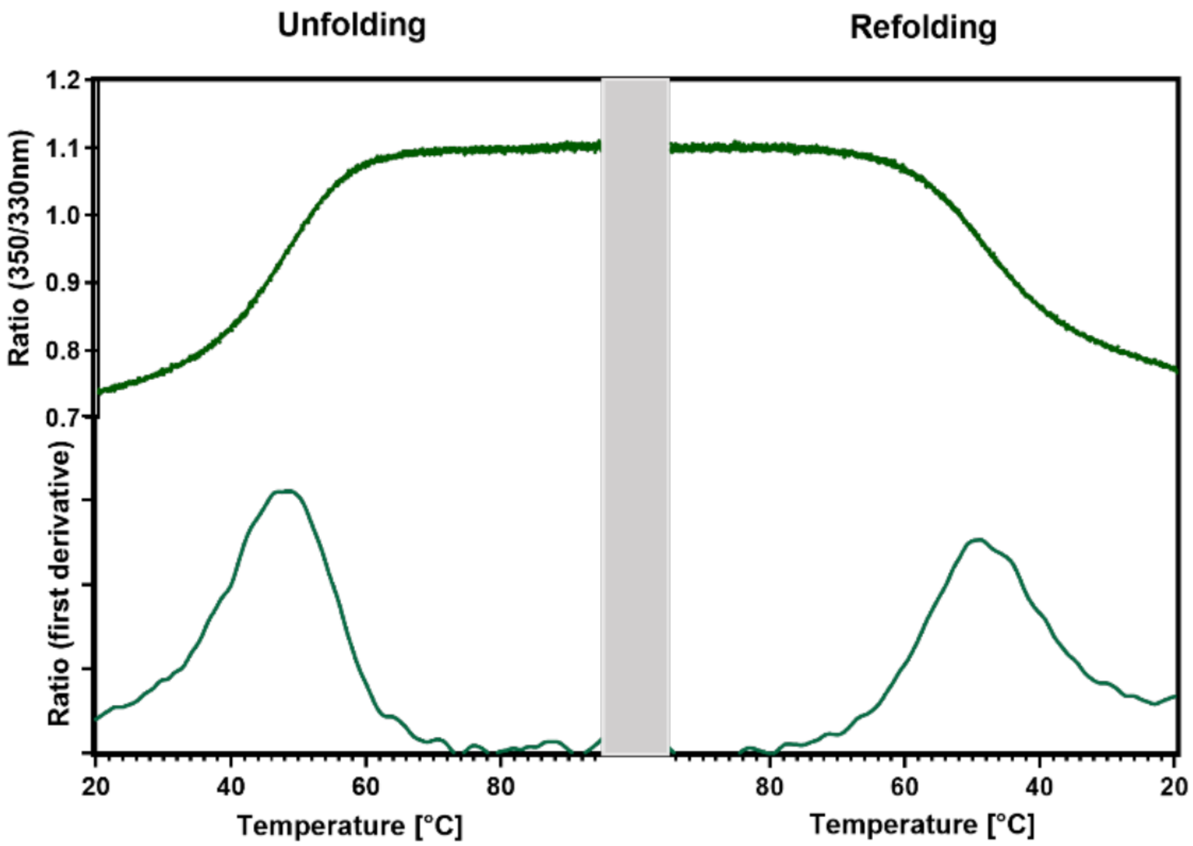

Supplemental Figure 12 │ Melting curve for SOX11-HMG

Thermal melting and refolding of SOX11 HMG domain. Upper, the ratio of tryptophan florescence (350/330) nm is plotted as a function of temperature. Lower first derivative of the unfolding and refolding profile. The calculated Tm is 48.5 ºC.

SUPPLEMENTAL METHODS

Doxycycline-induced expression of SOX11 in JVM-2

The JVM2 inducible cell lines were developed under the manufacturer's guidelines using the plasmids of the Retro-X Tet-On Advanced Inducible Expression kit (Clontech Laboratories). JVM-2-Tet-On cells were stable transduced with retroviral particles, carrying a plasmid containing either SOX11 under doxycycline control (pRetroX-TRE3G/hSOX11; human SOX11 cloned in pRetroX-TRE3G vector between ApaI and BamHI restriction sites) or empty plasmid (pRetroX-TRE3G), generating the JVM-2^{iSOX11} and JVM-2^{vector} cell lines, respectively. pRetroX-TRE3G/hSOX11 construct was validated by sequencing. JVM-2^{iSOX11} and JVM-2^{vector} cell lines were selected with 0.4 μg/ml puromycin (Sigma-Aldrich) for one week. The expression of SOX11 was induced by growing the cells in RPMI 1640 medium containing 0.1μM doxycycline (D9891, SIGMA) for 24 hours.

Cytarabine (ara-C) treatment

In our experiments, cells were incubated with ara-C for 72 h. For combining Vpx-mediated depletion of SAMHD1 to cytarabine treatment, 10^7 cells were incubated with 10 μL of VLPs for 3 h followed by cytarabine treatment for 72 h at 37°C and 5% CO_2. When ectopic expression of SOX11 was coupled to ara-C treatment, the ara-C treatment was performed after 24 h of inducing SOX11. Upon combining ectopic expression of SOX11 with Vpx-mediated depletion of SAMHD1, cells were cultured in doxycycline-supplemented RPMI 1640 for 24 h. The following day, ara-C treatment was applied after 3 h of Vpx treatment. To assess the response of JVM-2^{iSOX11} induced by different concentrations of doxycycline to cytarabine applied to JVM-2^{vector} and JVM-2^{iSOX11} for 72 h.

Cell cycle analysis

Analysis of the cell cycle was performed using the propidium iodide (PI) flow cytometry kit (ab139418, Abcam, Netherlands), according to the manufacturer's protocol. Briefly, $2x10^5$ cells were collected at specific time points, washed twice with PBS and fixed with 66% ethanol. Fixed cells were kept at 4°C until the last time point, and minimum 2 h before incubation with 1x Propidium Iodide plus RNase staining solution for 20 minutes at 37°C. Tubes containing the cells were then placed on ice until flow cytometry analysis using CytoFlex and the software FlowJo V10. A total of $1x10^4$ cells were recorded for each sample, singlets and live cells were selected and the amount of PI staining per cell was assessed, displaying the number of cells in the different cell cycle phases. Statistical analysis was performed using GraphPad Prism 9.

Heterotopic JVM-2 animal model

On day 0, 64 mice were randomly assigned into two groups for cell injections: those receiving 10^6 JVM-2^{iSOX11} cells (n = 32) and those receiving 10^6 JVM-2^{vector} cells (n = 32). Cells were injected subcutaneously on the flank in a 1:1 mixture of cells and Matrigel (Corning, 354234) in a total volume of 100 µL. On day 2, each group was randomly divided further into two groups: those receiving sterile water with 1 mg/mL Doxycycline (Sigma) (n = 16 for JVM-2^{iSOX11} and n = 16 for JVM-2^{vector}) and those receiving water without antibiotics (n = 16 for JVM-2^{iSOX11} and n = 16 for JVM-2^{vector}). On day 5, half of the mice from each group were randomly assigned to receive intraperitoneal injections of either ara-C (Cytarabine Accord, 100 mg/mL, Apoteket) 100 mg kg-1 (n = 8 for JVM-2^{iSOX11} with Doxycycline water, n = 8 for JVM-2^{iSOX11} with normal water, n = 8 for JVM-2^{vector} with Doxycycline water, n = 8 for JVM-2^{vector} with normal water) or PBS (n = 8 for JVM-2^{iSOX11} with Doxycycline water, n = 8 for JVM-2^{iSOX11} with normal water, n = 8 for JVM-2^{vector} with Doxycycline water, n = 8 for JVM-2^{vector} with normal water) consecutively for a total of 7 days. See Supplemental figure 8 for an overview of the groups. Tumour sizes were measured using calipers 3-5 times per week, and volume was estimated as tumour volume = 0.5 × tumour length × (tumour width).[1] General

welfare was monitored daily, and animals were weighed at least two times per week. Mice were euthanized when tumour volume reached 1,500 mm^3 or length reached 20 mm. In a handful of cases, animals were euthanized due to tumour ulceration, which was determined to be severe after consulting with the veterinarian. Upon autopsy, tumours were excised and split into two equal parts for either flash-freezing with liquid nitrogen or fixation in 4% formaldehyde before further processing. Fixed tumours were paraffin-embedded, formalin-fixed paraffin-embedded (FFPE) tissue blocks were cut using a microtome, and 4 μm-thick tissue slices were mounted on glass microscopy slides before deparaffination and rehydration using standard techniques. H&E staining was performed using standard procedures. For immunohistochemistry, antigen retrieval was following the protocol for UltraVision LP Detection System, HRP Polymer & DAB Plus Chromogen (Thermo Scientific). Slides were incubated overnight at 4°C with either 1:25 dilution of anti-SOX11 antibody (382M-14, Cell Marque) or 1:500 dilution of anti-SAMHD1 antibody (A303-691A, Bethyl) in 1% BSA in PBS. Slides were visualized using a Zeiss light microscope.

Co-IP and mass spectrometry

I. Co-immunoprecipitation

Granta519 and JeKo1 cells were cross-linked (2 x 10^7 cells per sample) in 11 % formaldehyde solution (11 % formaldehyde, 0.1 M NaCl, 1 mM EDTA, 0.5 mM EGTA, 50 mM Hepes, pH 8) for 8 min at RT. The reaction was quenched by adding 1/20 volume of 2.5 M glycine to the culture flasks after which cells were collected, washed 3 times in ice-cold PBS and re-suspended in hypotonic Lysis Buffer 1 (LB1, 10 mM Hepes-KOH, 10 mM KCl, 1.5 mM $MgCl_2$, 0.5 mM DTT, pH 7.5) for 10 min at 4 °C on a rotating platform. Nuclei was pelleted at 2500 rpm, 3 min at 4°C and re-suspended in Lysis Buffer 2 (LB2, 10 mM Hepes-KOH, 100 mM NaCl, 1 mM EDTA, 0.5 mM EGTA, 0.1% Na-Deoxycholate, pH 7.5). Re-suspended nuclear

fraction was sonicated in 6 cycles of 30 sec with 1 min intervals at 40 watts and supernatant collected (samples spun down at 14K, 10 min at 4 °C). LB1 and LB2 were supplemented with 1X protease inhibitor cocktail (Sigma-Aldrich, St. Louis, MO).

For each sample, 100 µL of Protein G Dynabeads (Invitrogen, Novex) were used. Briefly, beads were maintained at 4 °C, collected on a magnetic rack and re-suspended in fresh 0.5 % (w/v) BSA/PBS 3 times. Pre-blocked beads were incubated on a rotating platform at 4 °C O/N with 10 µg of anti-SOX11 (HPA000536, Sigma) or anti-IgG (Normal Rabbit IgG #2729, Cell Signaling Technology) in 250 µL 0.5 % (w/v) BSA/PBS. Antibody-saturated beads were washed 3 times in 0.5 % (w/v) BSA/PBS and re-suspended in 100 µL 0.5 % (w/v) BSA/PBS before added to the cell supernatant. The immunoprecipitation was carried out at 4 °C, O/N, on a rotating platform.

II. Sample Preparation for Mass Spectrometry

Proteins from three biological replicates of Jeko-1 and Granta-519 cells were immunoprecipitated using anti-SOX11 and IgG controls as described above. Samples were washed 4 times with wash buffer 1 (WB1, 50 mM HEPES, pH 7.6, 1 mM EDTA, 0.1% Na-deoxycholate), 2 times with wash buffer 2 (WB2, 50mM Hepes pH 7.6), and 2 times with wash buffer 3 (WB3, 20 mM ammonium bicarbonate NH_4HCO_3). Magnetic beads were released from the magnet and shaken during each wash. Proteins on beads were digested overnight with 10 µg/mL trypsin (Promega, V511) in WB3 at 37°C. Peptides were dried by speedvac and dissolved in 3% acetonitrile (ACN), 0.1 % formic acid before analysis on MS.

III. LC-ESI-LTQ-Orbitrap Analysis

Before analysis on the LTQ Orbitrap Velos (Thermo Fischer Scientific,), peptides were separated using an Agilent 1200 nano-LC system. Samples were trapped on a Zorbax 300SB-

C18, and separated on a NTCC-360/100-5-153 (Nikkyo Technos Ltd) column using a gradient of A (3% ACN, 0.1% FA) and B (95% ACN, 0.1% FA), ranging from 3 % to 40% B in 180 min with a flow of 0.4 µL/min. The LTQ Orbitrap Velos was operated in a data dependent manner, selecting 5 precursors for sequential fragmentation by CID and HCD (Higher-energy Collisional Dissociation), and analyzed by the linear iontrap and orbitrap, respectively. The survey scan was performed in the Orbitrap at 30,000 resolution (profile mode) from 300-2000 *m/z,* using lock mass at *m/z* 445.120025, with a max injection time of 500 ms and AGC set to 1 x 10^6 ions. For generation of HCD fragmentation spectra, a max ion injection time of 500 ms and AGC of 5 x 10^4 were used before fragmentation with 325% normalised collision energy. For FTMS (Fourier Transform Mass Spectrometry) MS2 spectra, normal mass range was used, centroiding the data at 7500 resolution. Peptides for CID were accumulated for a max ion injection time of 200 ms and AGC of 3 x 10^4, fragmented with 35% collision energy, wideband activation on, activation q 0.25, activation time 10 ms before analysis at normal scan rate and mass range in the linear iontrap. Precursors were isolated with a width of 2 *m/z* and put on the exclusion list for 90 s. Single and unassigned charge states were rejected from precursor selection.

IV. Peptide and Protein Identification

All Orbitrap data was searched by SequestHT under the software platform Proteome Discoverer 1.4 (Thermo) against the Uniprot human database (140407) and filtered to a 1% false discovery rate (FDR). A precursor mass tolerance of 10 ppm, and product mass tolerances of 0.02 Da for HCD-FTMS and 0.36 Da for CID-ITMS (Ion trap mass spectrometry) were used. Further settings used were trypsin with 2 missed cleavages, iodoacetamide on cysteine, and oxidation of methionine as variable modification.

Immunocytochemistry and proximity ligation assay

Immuno-fluorescence staining was performed on 4% paraformaldehyde-fixed and 0.1% Triton-X100-permeabilized cells on Superfrost Plus^{TM} Adhesion slides (ThermoFisher), followed by confocal microscopy imaging. Duolink in situ proximity ligation assay (PLA) was performed using the kit DUO92008 (Merck). Blocked fixed and permeabilized cells were immuno-stained with two antibodies raised in different species against SOX11 and SAMHD1 (listed in Supplemental table 1) in pre-heated humidity chamber at 37ºC for 1 h. The cells were then incubated with diluted PLA probes (PLUS and MINUS) that matches to host organism wherein primary antibodies are raised (DUO92004 & DUO92002, Merck). Subsequently, ligation of the probes and polymerization of the circular DNA were performed using DUO92008, (Merck) according to producer´s protocol. The efficiency of PLA was tested by visualizing fluorescent foci upon staining MCL cells using two antibodies raised in two different host organisms against the same protein, either SOX11 (Supplemental Figure11A) or SAMHD1 (Supplemental Figure 11B) as positive controls.

Cellular Thermal Shift Assay (CETSA)

To assess the shift of thermal aggregation temperature of SAMHD1 upon SOX11 overexpression, $1x10^6$ JVM-2^{vector} and JVM-2^{iSOX11} cells were collected and resuspended in 60 µL of TBS buffer (pH 7.5). Cell suspensions were heated at a range of temperature 38, 42, 48, 52, 55 and 60°C for 3 min, followed by 3 min incubation at room temperature. Cells were then lysed by 3 times of freezing/thawing cycles. Each cycle was comprised of 3 min on dry ice followed by 3 min incubation in water bath at 37°C. Total protein was quantified by Bradford assay and the procedure of Western blot was performed as described above. Band intensities of SAMHD1 at the different conditions were normalised to the respective band intensities of the thermostable Superoxide dismutase-1 (SOD-1) and the percentage of remaining proteins were

calculated and plotted to sigmoidal Boltzmann curve using GraphPad software (La Jolla California, USA).

Microscale Thermophoresis

Binding of SOX11 HMG to SAMHD1 was measured using Microscale Thermophoresis (MST). Experiments were performed in 25mM Hepes (pH8.0), 150mM NaCl, 5mM $MgCl_2$, 5mM DTT, 0.02% tween-20, and 0.1mg/mL BSA in standard capillaries on a Monolith NT.115. Fluorescence was observed using the Nanotemper His-Tag Red-tris-NTA at 25 nM bound to SAMHD1H215A(1-626) at 100 nM for 10 seconds at 80% MST power and 80% LED power.

I. Protein Expression

SAMHD1H215A (1-626) was cloned into a pET19b vector and expressed in *E. coli* BL21(DE3) gold cells as described in that study.[2] Purification was performed using NiNTA superflow (5 mL) column, followed by size exclusion chromatography on a Superdex 200 16/600 column equilibrated in in 20mM Tris-HCl (pH8.0), 150mM NaCl, 5mM MgCl2, 0.5mM TCEP.

SOX11 HMG domain (49-117) was cloned into a pET151 vector. Protein was expressed in *E. coli* BL21(DE3) Arctic-express cells at 10°for 20 h. Initial purification was performed in 8M urea over NiNTA superflow (5 mL) column followed by refolding and TEV protease treatment overnight at 4°C in 20mM Tris-HCl (pH8.0), 150mM NaCl, 0.5mM EDTA, and 0.5mM TCEP. After proteolysis, size exclusion chromatography was performed using a Superdex 75 10/300 increase equilibrated in 20mM Tris-HCl (pH8.0), 150mM NaCl.

II. SOX1 HMG Thermal melting

Reversibility and refolding of SOX11 HMG was confirmed using Prometheus NanoDSF. Thermal melting was followed by tryptophan fluorescence over a temperature range of 20°C to 95°C and refolding from 95°C to 20°C (Supplemental Figure 12).

Confocal imaging

Imaging was performed using a Nikon A1R confocal laser scanning microscope equipped with an inverted microscope Nikon Eclipse Ti-E. Laser lines used were 405 nm (DAPI), 488 nm (FITC) and 561 nm (TRITC). Images were captured with the imaging software NIS-Elements version 5.30.02. Fluorophores used in this study were FITC and TRITC and DAPI and TEXAS RED. The aperture size (pinhole) of objective lenses was set at 1.2. Images were captured at a magnification of 100X for PLA.

Image analysis

Colocalization of SOX11 or SAMHD1 by dual colour immunofluorescence was estimated using two channels, each representing one of the two molecules. Using ImageJ software (version 2.0), the output estimate of colocalization was calculated as the Pearson correlation (R).

Analysis of the images obtained from PLA was performed using CellProfiler (version 4.07) Pipelines were created in CellProfiler to determine mean number of foci per cell nucleus and mean fluorescence intensity per cell, as an estimate of the SOX11-SAMHD1 interaction. For analysing PLA images, the mean number of cells used for the analysis was 350 cells per biological replicate, and the analysis was performed on three independent biological replicates. We also created pipelines by CellProfiler to calculate percentage of immunofluorescent SOX11-positive cells and mean fluorescence intensity/cell at different concentrations of doxycycline. A mean of 750 cells were analysed per condition per replicate.

Statistical analysis

Comparison between groups representing different conditions of treatment and expressions of SOX11 or SAMHD1 was performed by two-tailed, unpaired student t-test with Welch

correction. For comparing different conditions of expressions status of SOX11 or SAMHD1 coupled to cytarabine treatment, Two-way ANOVA was performed so that expression status was set as column factor, while cytarabine dose (μM) was set as row factor. The accepted error value was <5%, that the cut-off value of the level of significance was set as ($P < 0.05$). The experiments were conducted on at least two independent biological replicates and the data were represented as Mean ± SEM with 95% confidence intervals. The dose response curves were charted by log transforming cytarabine concentration as X and relative viability values as Y, and IC50 values were calculated by performing Non-linear regression. Spearman rank correlation was performed to analyse correlation between SOX11 relative expression and percentage of SOX11-positive cells.

REFERENCES

1 Zittoun, R., Marie, J. P., Delanian, S., Suberville, A. M. & Thevenin, D. Prognostic value of in vitro uptake and retention of cytosine arabinoside in acute myelogenous leukemia. *Semin Oncol* **14**, 269-275 (1987).

2 Morris, E. R. *et al.* Crystal structures of SAMHD1 inhibitor complexes reveal the mechanism of water-mediated dNTP hydrolysis. *Nat Commun* **11**, 3165, doi:10.1038/s41467-020-16983-2 (2020).
